# What are the ways in which social media is used in the context of complementary and alternative medicine in the health and medical scholarly literature? a scoping review

**DOI:** 10.1186/s12906-023-03856-6

**Published:** 2023-02-02

**Authors:** Jeremy Y. Ng, Natasha Verhoeff, Jeremy Steen

**Affiliations:** Department of Health Research Methods, Evidence, and Impact, Faculty of Health Sciences, McMaster University, Michael G. DeGroote Centre for Learning and Discovery, Room 2112, 1280 Main Street West, Hamilton, ON L8S 4K1 Canada

**Keywords:** Complementary and alternative medicine, Social media, Social networks, Scoping review

## Abstract

**Background:**

Despite the increased use of social media to share health-related information and the substantial impact that complementary and alternative medicine (CAM) can have on individuals’ health and wellbeing, currently, to our knowledge, there is no review that compiles research on how social media is used in the context of CAM. The objective of this study was to summarize what are the ways in which social media is used in the context of CAM.

**Methods:**

A scoping review was conducted, following Arksey and O’Malley’s five-stage methodological framework. MEDLINE, EMBASE, PsycINFO, AMED, and CINAHL databases were systematically searched from inception until October 3, 2020, in addition to the Canadian Agency for Drugs and Technology in Health (CADTH) website. Eligible studies had to have investigated how at least one social media platform is used in the context of a single or multiple types of CAM treatments.

**Results:**

Searches retrieved 1714 items following deduplication, of which 1687 titles and abstracts were eliminated, leaving 94 full-text articles to be considered. Of those, 65 were not eligible, leaving a total of 29 articles eligible for review. Three themes emerged from our analysis: 1) social media is used to share user/practitioner beliefs, attitudes, and experiences about CAM, 2) social media acts as a vehicle for the spread of misinformation about CAM, and 3) there are unique challenges with social media research in the context of CAM.

**Conclusions:**

In addition to social media being a useful tool to share user/practitioner beliefs, attitudes, and experiences about CAM, it has shown to be accessible, effective, and a viable option in delivering CAM therapies and information. Social media has also been shown to spread a large amount of misleading and false information in the context of CAM. Additionally, this review highlights the challenges with conducting social media research in the context of CAM, particularly in collecting a representative sample.

## Background

Over 3.6 billion people worldwide used social media in 2020 [1]. This number has been predicted to increase to 4.41 billion by 2025. The American population using at least one social media platform such as Facebook, Snapchat, Instagram, Twitter or YouTube, has continuously increased over the past 15 years from just 5% of Americans in 2005 to 72% of Americans in 2019 [2]. Similarly, in 2017, 94% of Canadian internet users had at least one social media account [3]. Social media is comprised of a complex ecology of networking sites and falls in the larger context of health communication [4, 5]. Social media has changed the landscape of health information by allowing for dialogic communication rather than one-sided communication from health professionals and experts, resulting in health communicators such as practitioners, policy makers, and patients monitoring, listening to, and engaging with dialogue on social media [6–9]. It has been shown that 72% of internet users search for health information online and social media is one source of such health information [10, 11]. Social media is used to discuss health information with regards to complementary and alternative medicine (CAM) [11, 12]. How social media is used in the context of CAM would be valuable to better understand as surveys conducted by Pew Research Center have found that 35% of internet users have looked online for information about CAM specifically [11, 12].

CAM is frequently used across the world and consists of a variety of health care approaches that are not typically part of conventional medicine or completely integrated into the country’s main health care system. CAM includes, but is not limited to, manual therapies such as chiropractic and osteopathy, natural products such as herbal medicines and dietary supplements, and other forms of therapies including naturopathy, homeopathy, and traditional Chinese medicine [13–16]. The National Center for Complementary and Integrative Health (NCCIH) in the United States defines “complementary” approaches as those that are used together with conventional medicine and “alternative” approaches as those that are used in place of conventional medicine [13, 14, 17]. Positive motivations for trying CAM which may have contributed to its popularity include factors such as its accessibility, holistic and non-invasive nature, and perceived effectiveness and safety, while negative motivations include factors such as dissatisfaction with conventional medicine, rejection of science and technology, and desperation [18–21]. How CAM is portrayed in social media is important considering the ever-growing popularity and usage of social media and its ability to influence health behaviours and beliefs [22]. In the context of CAM, social media can be used to enhance patient’s access to health care related resources and support [23, 24]. Media sharing platforms such as YouTube are usually free, easy to use, and accessible on both mobile and desktop devices [25]. Also, unlike health information in the medical literature, when health information is shared on social media it is often written in lay terms [24, 26]. By allowing individuals to engage, interact with, and contribute health information, social media creates an environment that encourages patient conversation [27–29]. Sharing health information on social media can motivate and inspire others, but it also has the power to facilitate the spread of misinformation about health-related topics [30]. There are various scholars who study the processes and risks of misinformation and disinformation on social media and features of social media that may contribute to the spread of health-related misinformation [31–33]. Firstly, the low cost of generating and disseminating information over social media allows misinformation to spread globally at a rapid pace. Additionally, virtually anyone can post about CAM on social media regardless of academic or professional training, knowledge or skills [34]. Furthermore, it can be difficult to determine the credibility of social media content as users are self-publishers and often are not subject to scrutiny or accountability [30]. Moreover, since social media feeds are personalized to individual beliefs, values, preferences and biases, there is information silo and echo chamber effects which result in decreased exposure to differing opinions, reinforcement of confirmation biases, and the amplification of misinformation [35, 36].

Currently, to our knowledge, there is no review that compiles research on the ways in which social media is used in the context of CAM. Due to the increased impact of social media as a form of information sharing in North America, and the significant impact that CAM can have on people’s health and lives, it is important that a scoping review is performed to outline the research on this topic and identify the gaps. The results from this scoping review could help inform various stakeholders such as clinicians, policy makers, patients, and researchers. Thus, the aim of our scoping review is to provide a summary of the research on the ways in which social media is used in the context of CAM.

## Methods

### Approach

As described above, to our knowledge, there is a lack of review articles on the ways in which social media is used in the context of CAM. Thus, a scoping review methodology was appropriate as it allows for systematic scoping of a broad array of research and the identification of literature gaps [37]. The method for conducting this scoping review was based on Arksey and O’Malley’s five-stage scoping review framework [38]. This method was also supplemented by modifications proposed by Levac, Colquhoun, & O’Brien and Daudt, van Mossel, & Scott [39, 40]. We used this five-stage scoping review framework to ensure that all scoping review prerequisites were met including identifying and analyzing the current literature on the topic, summarizing it, and recognizing knowledge gaps that could potentially be looked into by future research [40].

#### Step 1: Identifying the research question

Our research question is as follows: what are the ways in which social media is used in the context of CAM in the health and medical scholarly literature? For the purpose of this scoping review, we referred to the Cochrane Complementary Medicine group’s operational definition of CAM [41]. For social media, we referred to the definition by Obar et al. 2015 as it is comprehensive, containing four parts, and has been used by many others in the academic community [42]. This definition states that social media consists of the following four main characteristics:

- 1. Social media services are (currently) applications that are Web 2.0 Internet-based
- 2. The lifeblood of social media is user-generated content
- 3. For a site or app designed and maintained by a social media service, individuals and groups create user-specific profiles
- 4. The development of social networks online by connecting a profile with those of other individuals and/or groups is facilitated by social media services

#### Step 2: Finding relevant studies

After identifying the research question, we found relevant studies to include in our scoping review using a comprehensive and systematic search strategy. We searched the bibliographic databases MEDLINE, EMBASE, PsycINFO, AMED, and CINAHL. Indexed headings and keywords relating to social media and CAM were used in each of the search strategies where appropriate. Additionally, we searched the Canadian Agency for Drugs and Technology in Health (CADTH) for any grey literature related to our topic. Search terms on CADTH included “complementary and alternative medicine” and “social media”. The search of these various databases and websites included literature from inception until October 3, 2020. A sample search strategy used is shown in Table 1.

**Table 1:** MEDLINE Search Strategy for Studies Investigating How Social Media is Used in the Context of CAM, Executed October 3, 2020

Database: OVID Medline Epub Ahead of Print, In-Process & Other Non-Indexed Citations, Ovid MEDLINE(R) Daily and Ovid MEDLINE(R) 1946 to Present Search Strategy:
––––––––––––––––––––––––––––––––––––––––
1 (alternative medicine* or alternative therap*).mp. (24,388)
2 (complementary medicine* or complementary therap*).mp. (22,183)
3 exp Complementary Therapies/ (228,775)
4 (integrat* adj1 (medicine or therap*)).mp. (4385)
5 exp Integrative Medicine/ (1582)
6 naturopath*.mp. (1597)
7 exp Naturopathy/ (999)
8 acupunctur*.mp. (29,450)
9 exp Acupuncture Analgesia/ or exp Acupuncture Points/ or exp Acupuncture Therapy/ or exp electroacupuncture/ or exp Acupuncture/ (25,426)
10 (chiropract* or spinal manipulation*).mp. (8728)
11 exp Chiropractic/ or exp Manipulation, Chiropractic/ (4104)
12 (herb* adj1 (medic* or therap* or supplement*)).mp. (26,197)
13 exp Medicine, East Asian Traditional/ or exp Medicine, Chinese Traditional/ or exp Herbal Medicine/ or exp Plants, Medicinal/ or exp Phytotherapy/ (109,867)
14 tcm.mp. (11,027)
15 exp Drugs, Chinese Herbal/ (43,922)
16 traditional Chinese medicine.mp. (21,324)
17 exp Medicine, Ayurvedic/ (2231)
18 ayurved*.mp. (6714)
19 acupressure.mp. (1459)
20 exp Acupressure/ (758)
21 applied kinesiolog*.mp. (99)
22 exp Kinesiology, Applied/ (312)
23 herbalism.mp. (152)
24 exp Osteopathic Medicine/ or exp Manipulation, Osteopathic/ (4050)
25 osteopath*.mp. (7627)
26 exp Mind–Body Therapies/ (50,683)
27 mind–body*.mp. (5356)
28 exp Yoga/ (2857)
29 yoga.mp. (5708)
30 or/1–29 (373,277)
31 exp Social Media/ or exp Social Networking/ or exp Blogging/ (12,139)
32 (((e or electronic) adj3 newsletter*) or ((peer to peer adj5 network*) or p2p) or (digital adj5 platform*) or (forum* adj3 (internet or web* or chat*)) or (rss adj3 (reader* or feed*)) or (social adj3 media*) or (social adj3 medium*) or (social adj3 network*) or (twitter or tweet*) or (viral adj5 market*) or ("web 2.0" or "web 2") or (website* or "web site*" or webpage* or "web page*") or altmetric or badoo or blackplanet or blog* or buddypress or buzznet or care2 or classmates or clicktotweet or "content communit*" or couchsurfing or discord or facebook or faces or fiverr or flickr or flixster or fotki or fotolog or foursquare or gaiaonline or geni or girlsaskguys or googl* or groupme or hashtagify or hashtags or "health 2.0." or hi5 or hkgolden or hootsuite or i-phone* or instagram or keyhole or kik or kuaishou or "last.fm" or linkedin or lipstickalley or lithium or livejournal or "medicine 2.0." or meetup or microblog* or myspace or myspace or naijapals or neopets or ning or okcupid or pinterest or podcast* or qq or qzone or "really simple syndicat*" or reddit or sermo or skype or skyrock or smartphone* or snapchat or stumbleupon or tagged or telegram or tiktok or trendsmap or tumblr or tweepsmap or twitter or "user generated content" or weblog* or wechat or weibo or weixin or whatsapp or xing).ti. (65,844)
33 or/31–32 (71,355)
34 30 and 33 (465)
35 limit 34 to english language (411)
***************************

#### Step 3: Selecting the studies

We included research articles and protocols in this scoping review. While review articles were not eligible, we screened the reference lists of review articles that appeared relevant to our research question to identify eligible articles. Conference abstracts, commentaries, editorials, letters to the editor, opinion pieces, and articles that were not published in the English language were ineligible. Additionally, articles that could not be publicly accessed, found through our library system, or ordered via interlibrary loan were excluded. In order to be eligible, it had to be evident in the record’s title and/or abstract that the study was about how any form(s) of social media is used in the context of any form(s) of CAM. Two authors (JYN and NJV) pilot-screened a subset of titles and abstracts individually and then met to verify their application of the inclusion criteria. Then, all full articles were screened independently in duplicate by JYN and NJV. In the case of disagreement about article eligibility, when discussion between the two authors (JYN and NJV) was not sufficient to resolve the disagreement, a third author (JS) partook in the discussion and a majority vote took place to determine eligibility.

#### Step 4: Charting the data

Arksey and O’Malley’s descriptive narrative method was used to critically assess articles meeting the inclusion criteria [38]. To chart the eligible articles, the following information was extracted: first author and year of publication, country of authors, study setting, article type, objective, population and sample size, CAM discussed/used, social media discussed/used, primary and secondary outcomes, how primary and secondary outcomes were measured, main findings, challenges encountered, and study conclusions. Two authors (JYN and NJV) participated in a pilot data extraction exercise using a subset of eligible articles. Any discrepancies between the pilot data extraction of the two authors were discussed and resolved by three authors (JYN, NJV and JS). Then, data from all eligible articles was independently extracted by JYN and NJV; following this, all authors met to discuss and resolve any discrepancies. Only data relevant to the research question was extracted and charted from the eligible studies. Additionally, a descriptive map of the literature on our topic was created which highlighted key themes that emerged from the analysis.

#### Step 5: Collating, summarizing, and reporting the results

Tables were used to summarize charted data and an inductive thematic analysis was performed on descriptive data [43]. The descriptive data was reviewed by all authors. NJV and JS then identified codes for the descriptive data based on main topics discussed in the articles and organized the articles into thematic groups. The thematic groups grouped articles based on the identified commonly discussed topics. NJV and JS also created a narrative discussing how these results connect to the research question. Moreover, NJV and JS identified knowledge gaps in the current literature. JYN reviewed all of the aforementioned tasks, and any discrepancies were discussed and resolved by all authors.

## Results

### Search results

**Fig. 1:**
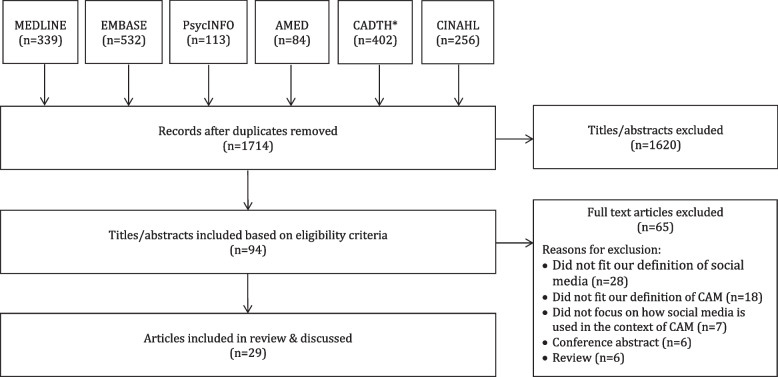
PRISMA Diagram. *List of Abbreviations: CAM = complementary and alternative medicine, CADTH = Canadian Agency for Drugs and Technologies in Health

Searches retrieved 1714 items following deduplication, of which 1620 titles and abstracts were eliminated, leaving 94 full-text articles to be considered. Of those, 28 were not eligible because they did not fit our definition of social media (e.g., newspapers or magazines), 18 did not fit our definition of CAM, 7 did not focus on how social media is used in the context of CAM, 6 were an abstract, and 6 were a review. This left 29 articles for inclusion in this scoping review [44–72]. In Fig. 1, a PRISMA diagram can be found depicting this process.

## Eligible article characteristics

Eligible articles were published from 2012 to 2020 and were conducted by researchers from the United States (*n* = 17), Canada (*n* = 4), Australia (*n* = 2), France (*n* = 1), Germany (*n* = 1), Spain (*n* = 1), and Taiwan (*n* = 1). Additionally, one study was conducted by researchers from China, Australia, and the United Kingdom (*n* = 1), and another study was conducted by researchers from Iraq and Jordan (*n* = 1). Of these 29 eligible articles, 10 focused on a study population from a single country, meaning that only social media content posted by users from a specified country was included in the study. These countries included the United States (*n* = 5), Australia (*n* = 1), Germany (*n* = 1), Iraq (*n* = 1), Spain (*n* = 1), and Taiwan (*n* = 1). The remaining 19 eligible articles focused on social media content from more than one country, 13 of which focused on an international sample of social media content (i.e., all of Twitter). While a diverse array of CAM was explored, the most common were yoga (*n* = 4), medicinal cannabis (*n* = 4), dance therapy (*n* = 2), music therapy (*n* = 2), and spinal manipulation (*n* = 2). While nutrition is not typically defined as CAM, we included articles such as the one by Chan et al. despite their focus on nutrition because they included CAM representation and discourse, such as the theme of promoting alternative medicine while challenging conventional medicine [46]. The most commonly discussed social media platforms were Twitter (*n* = 6), Facebook (*n* = 5), and YouTube (*n* = 4). The articles used a variety of qualitative and mixed methods in their social media research approaches. Of the 29 eligible articles, 24 were described generically as qualitative without naming a specific design or were described in terms of data collection techniques (e.g., focus group and interview) or analytic techniques (e.g., content analysis and discourse analysis). Of the remaining 5 eligible articles, two were identified by the authors as following a case study design, one was identified as following quantitative approaches, and two were identified as mixed methods studies based on its methodology and the presence of a combination of qualitative and quantitative approaches. The details associated with all eligible article characteristics, including study aims, can be found in Table 2; the main findings, challenges encountered, and conclusions of all eligible studies can be found in Table 3. No studies reported any secondary outcomes.

**Table 2:** General Characteristics of Eligible Studies

First Author and Year	Country of Authors	Study Setting	Article Type	Objective	Population and Sample Size	CAM Discussed/Used	Social Media Discussed/Used	Primary Outcomes	How Primary Outcomes Were Measured
Al-Samaray et al. 2020 [44]	Iraq and Jordan	Iraq	Qualitative study	To evaluate the impact of social media on consumers' decisions to use dental products made primarily from herbal ingredients rather than well-known chemical formulas	*n* = 300 (*n* = 100 pharmacists, *n* = 100 dentists and *n* = 100 consumers)	Herbal dental products	Social media (not specified), but Facebook and Twitter mentioned in results	Consumers' interview answers	Face to face interviews
Barnes et al. 2020 [45]	Australia	Australia	Case study	To describe how Facebook was used to recruit a targeted sample of expectant and breastfeeding mothers to participate in a national online survey	1418 participants enrolled in the study, 810 completed the 20-min (70 question) survey	CAM	Facebook	Recruitment post success and response rates	Reach, impressions, link clicks, post clicks, and click through rates were used to determine the success of the post, as well as the cost per day and cost per engagement with the post for boosted posts. Data from the Facebook ‘Insights' page was used to calculate response rates in a variety of ways
Chan et al. 2020 [46]	United States	International	Content analysis	To contrast and compare the nutrition advice given by registered dieticians (RDs) and non-RD bloggers on blogs	*n* = 400 blog posts	Nutrition	Blogs	Codes and themes from each blog post's coding; Readability of RD and non-RD blog posts using the Simple Measure of Gobbledygook (SMOG)	To minimise error, all posts in the sample were printed and given identification codes for each researcher to use in their analysis. After a lengthy analysis process, each post was subjected to a text-based content analysis using open coding to identify trends in the data and develop themes. A Simple Measure of Gobbledygook (SMOG) readability measure was used to assess the differences in readability between RD and non-RD blogs based on themes found in RD and non-RD blogs, respectively
Gierth et al. 2020 [47]	Germany	Germany	Exploratory experimental study design	To look into the effects of science-critical user comments attacking Facebook posts that made scientific claims	There were 2 smaller studies in the larger study: 1. 144 participants (98 were female), median age was 22 years, 84% were students 2. 160 participants (114 of which were female), median age was 23 years, 80% were students	Homeopathy	Facebook	Participants' attitudes toward and self-reported knowledge and involvement in the topics discussed in the Facebook posts; their agreement with the research claims, perceived study credibility, agreement with the user comment with one item each, and source trustworthiness judgement	Questionnaires with five 5-point Likert-type scale items for each topic to determine participants' attitudes toward the topics were used. Single items to assess self-reported knowledge and involvement in each subject were also used. The Muenster Epistemic Trustworthiness Inventory was used which assesses three aspects of a scientist's trustworthiness: expertise, benevolence, and integrity. It has a total of fourteen 7-point semantic differentials, six of which make up the expertise scale and four make up the benevolence and honesty scales, respectively
Kawchuk et al. 2020 [48]	Canada, Denmark and Switzerland	International (all of Twitter)	Content analysis	To detail how Twitter activity can be used to discuss misinformation claiming a link between spinal manipulation therapy and increased immunity	1118 Tweets were generated from search one, 778 of which were not relevant, 187 were promoting SMT for immunity, 141 refuting and 12 neutral, 809 tweets didn't mention a professional, 7 mentioning a professional irrelevant to the study	Spinal manipulative therapy (SMT)	Twitter	Tweet mentions (tweets with search terms) over time, tweet coding and sentiment (emotional expression), themes and word frequency, profession coding (professions mentioned), influencers (tweet authors with an engagement score greater than zero), and tweet author demographics	The primary search (Search #1) was built using Boolean syntax and consisted of three main components. Four evaluators scored this sample of 86 tweets independently to calibrate their use of the Twitter Tone Index (TTI). The TTI was then used by these same evaluators to independently assess each tweet resulting from Search #1. Tweets with less than three evaluators in agreement were discussed and a fifth evaluator broke any unresolved ties. Importantly, only whether tweets mentioned a profession could be coded; it was not possible to determine whether or how the author was associated with a specific profession
Merten et al. 2020 [49]	United States	International (Pinterest)	Content analysis	To see how cannabidiol (CBD) products were presented on Pinterest	226 pins of the 1280 cannabidiol and CBD related pins were analyzed	Marijuana (CBD)	Pinterest	Pin coding and engagement	Using code categories from previous health-related Pinterest studies, a codebook for CBD products was developed, tested, and used for this research. A pilot set of 58 Pinterest pins was used to test the codebook. Researchers independently coded the pins before meeting to address coding inconsistencies and challenges. Engagement was measured using previous studies' methods, which included the amount of saves (formerly known as repins) and comments
Allem et al. 2019 [50]	United States	International (Twitter)	Content analysis	To quickly capture and describe the public's recent experiences with cannabis using publicly accessible data from Twitter	*n* = 60 861 tweets	Cannabidiol (CBD)	Twitter	Topic classification of the tweets	Based on the presence of at least one topic-related pattern, each tweet was assigned to one or more topics. This was done by examining each tweet for the presence of a specified set of patterns representing a topic using a rule-based classification script written in Python
Aubrey et al. 2019 [51]	United States	United States	Experimental study	To see how self-objectification, appearance anxiety, and product preferences are affected by how health advice for adolescent girls is framed	154 girls	Yoga	YouTube	Girls' self-stated thoughts on self-objectification, appearance anxiety, and preference for appearance-enhancing products	Self-objectification and having participants describe themselves using 10 words of a 40-word bank. Attempted to balance negative and positive descriptors in each category. Used the Trait Self-Objectification Questionnaire (described in the “Trait Self-Objectification” section, *r* = .25, *p* = .002). Study also looked at appearance anxiety and preference for appearance-enhancing products
Cano-Orón 2019 [52]	Spain	Spain	Content analysis, qualitative discourse analysis and in-depth interviews	To investigate the discourse of Spanish sceptics on Twitter and understand the mobilisation against these therapies by the sceptical movement	6252 tweets from the #StopPseudociencias campaign were identified and 912 of these were used as the basis of the content analysis	CAM	Twitter	Analysis of tweets and discourse related to CT skepticism. Variables were created ad hoc using the casuistry of the campaign as a basis and looking into the standard of the other research	The different campaign hashtags’ tweets were collected with the data scraping tool data-miner.io. For the content analysis, the three variables tweets were analyzed on were (a) positioning (whether the tweet was sceptical, neutral or favourable to CTs, or whether it was irrelevant to the topic, (b) type of information sources contained in the messages and (c) use of mentions. Following this, a content analysis methodology was applied to the description that they put in their bios and the variables analyzed were (a) the account type (anonymous/corporate/personal), and, for those personal accounts, (b) the profession and (c) whether they were specialised in biomedicine. Manual content analysis and a qualitative discourse analysis of the tweets was also performed using Nvivo, in order to categorise the arguments raised by the users
Hasan et al. 2019 [53]	United Kingdom	Did not specify	Experimental, quantitative study	To evaluate the effectiveness of hypnotherapy on skype for IBS patients and draw a comparison of it with face-to-face treatment	*n* = 20	Hypnotherapy	Skype	IBS Symptom Severity	A minimum 50-point reduction in the IBS Symptom Severity Score (IBS-SSS)
Hu et al. 2019 [54]	International (China, United Kingdom, Australia, United States)	Did not specify (Online)	Content analysis/Comparative analysis	To look at the consistency of adverse events (AEs) and adverse drug reactions (ADRs) documented in the literature, as well as in monitoring and social media data	660 posts (351 Baidu post bar themes and 309 Sina micro-blogs)	Chinese patent medicine	Baidu post bar and Sina micro-blog	Safety related information about Chinese patent medicine on Baidu post bar and Sina	The contents of the post bar and blogs were screened by two reviewers separately, and agreements were reached. The reporter abstracted information relevant to protection, including manifestations, severity, length, effects, combined interventions, and causal-relation judgments. All of the information gathered was categorised as AEs. The ADR words are based on the following guidelines: 1) Names of AE/ADRs found in various resources that were compatible with WHO-ART words were specifically used; 2) For records where the AE/ADR names did not fit WHO-ART, the names were coded according to the thorough definition of the AE/ADRs; 3) for records where the names and descriptions of AE/ADRs did not match, descriptions were used as the key proof of coding; 4) for records with ambiguous names and descriptions where coding was difficult, the records were labelled as "unable to code" The frequencies and relative frequencies of AEs, ADRs, and the systems-organs groups they affect were counted. Each individual symptom or abnormality was counted when more than one AEs or ADRs occurred in the same patient. When a single AE or ADR was graded into two or more WHO-ART system-organ groups, it was counted multiple times. The top five AEs, ADRs, and affected system-organ groups were then ranked
Lacasse et al. 2019 [55]	Canada	Did not specify if they focused on posts of a specific country	Text, content, and image analysis	To investigate the themes and patterns that prominently emerge within the engagement (comments, conversations) from posts tagged #yoga on Instagram	Content: *n* = 35,000—Analysis of 100 unique authors and images from the unique data set	Yoga	Instagram	Netlytic categories; coding of themes by 2 researchers independently	To identify the most popular words in the dataset, Netlytic and content category analysis was used to determine the themes within the discussion. After identifying the terms, the prevalence of the use of the most popular words in the dataset was determined and recorded using a table. A pie chart was created to represent the four content categories and the prevalence of the words relating to these categories
Lognos et al. 2019 [56]	France	The posts were from a French database. Geographical information on whether the authors were French or Francophonie was not obtained	Exploratory study	By exploring posts published in health forums and French-language social media groups, to see whether patients with breast cancer were interested in CAM	*n* = 264,249 posts	CAM	Inpatients and Breast Cancer social forums; Facebook	CAM-related terms used in posts by cancer patients on health forums and social media groups, and how they fit into the five categories of NPIs: visual, nutritional, psychological, physical, and other	We used a database collected from internet-based French-language forums and discussion groups of patients treated or followed for breast cancer to perform a retrospective frequency analysis of the terms used in NPIs. With the consent of the French non-profit breast cancer patient association, the 264,249 posts published in these forums and Facebook pages (without additional details for each post, such as the number of views, comments, shares, or likes) were gathered and anonymized. In the compiled database of 264,249 articles, all NPI words were searched. We performed two descriptive frequency analyses: (1) an examination of the occurrences of NPI groups and their synonyms, and (2) a subcategory examination of NPI words, synonyms, and related terms (e.g., ingredient, technique, method, and profession)
Majmundar et al. 2019 [57]	United States	International (Instagram)	Content analysis	To identify the themes, user profiles, and references to different types of e-liquid solutions used with KandyPens from Instagram posts about KandyPens	*n* = 1775 Instagram posts from 546 unique accounts	Cannabis aromatherapy	Instagram	Thematically coded categories and references to e-liquid solutions of the posted images and captions; Instagram user profile categories associated with each post	Investigators collaborated to familiarise themselves with the data, then created a coding frame and identified common categories. The posts' themes were identified. Each Instagram user profile associated with each post was assigned to one of several categories
Plachkinova et al. 2019 [58]	United States	Not specified	Content analysis/mixed methods	To respond to the question, “Can a social media forum be used to boost CAM communication?” and explain the development of an online community to link patients with CAM and western biomedicine providers	Semi-structured interviews and user feedback: 12 key stakeholders from the CAM, biomedicine, and patient populations; Content analysis: based on content of 11 active users of the tool; Usability analysis: 8 graduate students	CAM	Social media tool created by the researchers	Usability, key concept codes from the semi-structured interviews, google analytics data, content analysis codes	We performed usability tests with eight subjects to assess the mobile application's technological effectiveness. Following the interviews, the participants were sent a link to an online survey in which they were asked to rate the tool's usability. The System Usability Scale was used (SUS). Semi-structured interviews were conducted with a total of twelve participants. Three people were patients, six were CAM or Integrative Medicine (IM) practitioners, and three were MDs or Physician Assistants working in a typical medical environment who conducted interviews
Rizvi et al. 2019 [59]	United States	International (Yahoo! Answers)	Content analysis	To use topic modelling to better understand consumer awareness needs for dietary supplements (DS) and to assess the accuracy of correctly identifying topics from social media	16,095 unique alternative medicine questions and 438 unique DS Ingredients	Dietary supplements	Yahoo! Answers	38 health-related categories and 12 higher level groups based on the Correlation Explanation topic modeling results	To uncover the different topics in which consumers are most interested, we used Correlation Explanation (CorEx), an unsupervised topic modelling tool. All 200 topics produced by CorEx had their keywords manually checked and assigned to 38 health-related categories, which corresponded to 12 higher-level classes
Webb et al. 2018 [60]	United States	International (Instagram)	Content analysis	To explore the shared and unique approaches used to encourage fitness and health on Instagram	*n* = 300	Yoga	Instagram	Coded attributes of the images taken from Instagram. The distribution of the coded attributes tagged from photos with #curvyyoga	Systematically coded images for factors such as: sociodemographic (e.g., race/ethnicity, gender), body-as-process (e.g., physically active portrayals), and body-as-object (e.g., weight loss, body modification) attributes
Yin et al. 2018 [61]	United States	International (YouTube)	Content analysis	To look at the different types of user-generated content on YouTube about cupping therapy for pain management	*n* = 100 videos	Cupping therapy	YouTube	Content and sources of the videos	Coded the 100 most widely viewed cupping videos for content and sources. Used logic regression to obtain the associations
Dai and Hao 2017 [62]	United States	All of Twitter (International) 78% of the tweets with an identifiable location were from the United States of America	Content analysis	To evaluate factors that could potentially impact the public's attitudes to PTSD related marijuana use on Twitter	*n* = 1,253,872 tweets	Marijuana (medicinal)	Twitter	Text mining, the opinions represented by the tweets measured through human judgement and the Naïve Bayes model, self reported location and the state regulation and socio-economic information associated with that location	Text mining was used to identify PTSD tweets related to marijuana use. Using a combination of human judgement and the Naïve Bayes model, the opinions represented by the tweets were measured (whether they support or are neutral/against marijuana use for PTSD). Geographical, state regulation, and socio-economic factor data was also collected. To obtain the location information from a large number of tweets, the following was extracted: geographic locations based on the self-reported location, time zone and longitude/latitude information. When determining state regulation, states were classified based on the following 3 groups: 1) legalized marijuana use for both medical and recreational use 2) legalized medical but not recreational use 3) no laws legalizing marijuana. The 2014 American Community Survey (ACS) was used to find socio-economic information. Then, the Spearman correlation was used to assess how state regulation and socio-economic factors correlate with the attitudes present in marijuana-PTSD tweets
Marcon et al. 2017 [63]	Canada	International (YouTube)	Content analysis	To explore debates or disputes about chiropractic in the comments section of popular YouTube chiropractic videos	*n* = 4 videos	Chiropractic manipulation	YouTube	Coding categories and analysis of the comments	Categories were assigned to the various comment characteristics and comments were coded from each top-10 list (likes and replies) for each of the four videos. Then, for the top-10 discussions in each of the four videos, we used coding analysis to determine the types of discussions that were taking place and whether debates were taking place. This method was guided by a classification scheme for YouTube comment content review and a YouTube interaction guide
Bitcon et al. 2016 [64]	Australia	International	Thematic analysis	To look for evidence of herbalism discussed in the blogosphere	*n* = 9 blogs	Herbalism	Blogs	Coded broad-themes and sub-themes	Over a four-week period, the post history of each of the final samples of blogs was collected. To identify bloggers' gender, age, location, level of education, and occupation, demographic data was gathered from their biographies and blog content. Next, a data analysis was carried out. The content of the blog was openly coded until the themes were exhausted. During the authors' regular meetings, implicit and explicit descriptions of stakeholder experience were extracted, and the coding was organized into broad themes and sub-themes
Guo et al. 2016 [65]	Taiwan	Taiwan	Program evaluation	To measure the effectiveness of a mindful yoga program that is delivered on social media to women that are pregnant	50 pregnant women	Mindful yoga	Facebook	Comments received via telephone from study participants to determine usage of Facebook and/or DVD program frequency and preference	A phone call was made to validate the participant's use of Facebook and/or the DVD software, as well as their opinions on it
Krampe et al. 2016 [66]	United States	United States	Program evaluation	To evaluate Fuze and compare it with Skype as a platform for dance-based therapy with older adults and nursing students	The participants from the assisted living facilitiy (ALF) were 14 residents (10 completed evaluations, with a ratio of 60% female to 40% male), with an average age of 77 years (range, 59–97 years), and with a diversified racial population (white, 60%; African American, 40%) Traditional sophomore baccalaureate nursing students (*n* = 14) from a Midwest university enrolled in a Health Promotion Across the Lifespan course participated in the Fuze dance therapy Four students were on site at the ALF; the other 10 were with a nursing instructor at the school of nursing (SON)	Dance-based therapy	Fuze (Skype was mentioned in discussion)	Program evaluation survey	The following major areas were evaluated through participant completion of a post-intervention form: (1) feasibility, including visual and audio quality, (2) engagement between the older adults, and (3) nursing students’ overall satisfaction
Marcon et al. 2016 [67]	Canada	International (Twitter)	Case study analysis	To see if there were any criticisms or debates about SMT's effectiveness and risks on Twitter	Sample (*n* = 1267) from corpus of a total of 20,695 tweets	Spinal manipulative therapy (SMT)	Twitter	Text content of the tweets and number of mentions or hashtags in each critical or skeptical tweet	Each critical or cynical tweet was counted for the number of mentions and hashtags. The prevalence of sceptical or critical tweets was then calculated by compiling lists of the top ten retweets and liked tweets from each corpus. Retweets and likes were used to determine how much coverage a tweet had received and which information was most commonly disseminated using Twitter tools
Robertson 2016 [68]	United States	United States	Quantitative study (experimental, longitudinal)	To determine the impact of a music therapy blog on Newborn intensive care unit (NICU) music therapy resources awareness, and education	12 participants: 5 neonatologists, 6 nurse practitioners, 1 physician assistant	Music therapy	Blog created by the researcher	(1) how informed the participants are about NICU music therapy therapy services; (2) how confident they are when ordering NICU music therapy services for patients; (3) how helpful they believe music therapy is for NICU patients; (4) how comfortable they are communicating about music therapy with other NICU staff; and (5) how useful they perceive the MT blog to be for obtaining information	The survey had five questions that measured the primary outcomes. All questions were rated on a 5-point Likert scale with descriptors such as "No Knowledge, Not Comfortable," "Moderate Knowledge, Moderately Confident," "Very Knowledgeable, Very Confident," and "Very Knowledgeable, Very Confident,". Using QualtricsTM, the researcher developed an online version of the questionnaire. Due to the primary care providers' time constraints, a 5-point Likert-type scale was used for a fast and effective response experience. The sensitivity index for this survey ranges from 5 to 25, with higher scores suggesting greater knowledge of NICU music therapy services. Before, during (after two weeks), and after the study finished, survey results were recorded
Keim-Malpass et al. 2013 [69]	United States	United States	Observation/thematic analysis	To look at descriptions of complementary therapy used by young women diagnosed with cancer who kept an online cancer blog	*n* = 16 blogs	CAM	Blogs	Descriptions of CAM used by young women diagnosed with cancer between the ages of 20 and 39 who kept a cancer blog online	Analysis of textual data and reflective journal entries and field notes
Krampe et al. 2013 [70]	United States	United States	Case study analysis	To describe how video call technology was used to introduce nursing students to a group of older adults during a dance-based therapy session	120 students but 8 were the focus (2 men and 6 women), and 5–6 older adults	Dance based therapy	Skype	Feedback about the process from all parties involved	An analysis of phone conference debriefings
Walden 2013 [71]	United States	Did not specify	In-dept interviews with bloggers and textual analysis of blogs	To determine the reasons for why naturopathic physicians utilize blogs	18 interviewees (1 naturopathic medical student and 17 naturopathic physicians) and the blogs of 14 of them	Naturopathy/Naturopathic medicine	Blogs	The reasons for why naturopathic physicians utilize blogs	Data analysis, coding of interviews, blog analysis (check of blog characteristics and a deep textual analysis of blog themes)
Gregory et al. 2012 [72]	United States	International (YouTube)	Descriptive Analysis	To see if music therapy documentary videos on the internet effectively and accurately represent the profession	*n* = 27 documentaries	Music therapy	YouTube	An examination of the rubric, which includes documentaries chosen by their creators, video-specific information, and therapy-specific information	Researcher-created rubric nearly identical to the rubric created for the earlier investigation of YouTube music therapy session videos

**Table 3:** Main Findings, Challenges Encountered, and Conclusions of Eligible Studies

First Author and Year	Main Findings	Challenges Encountered	Study Conclusions
Al-Samaray et al. 2020 [44]	Social media posts have played a key role in shifting customers' minds about using herbal products. Herbal dental products are commonly used by people of all ages and educational levels. When used appropriately for the targeted audience through the appropriate social network, social media will guide the audience toward a specific product	Not reported	Herbal dental products are commonly used by people of all ages and educational levels. When used appropriately for the targeted audience through the appropriate social network, social media will guide the audience toward a specific product
Barnes et al. 2020 [45]	After 10 weeks of recruitment, 1418 participants had enrolled in the study, and of these 810 (57.1%) completed the 20-min (70 question) survey. Women participated from across Australia. Both paid and purposive approaches to promotion contributed to recruitment success. Paid promotions at higher costs for fewer days were the most successful. Total paid promotion costs were (Australian) $1147.97 (or $1.44 per completed survey). Purposive promotion was slower, but also contributed significantly to the number of people who saw the posts and clicked through to the survey. Traditional response rate calculations showed a response rate of 0.8%. Using post clicks and survey link clicks in calculations, resulted in response rates of 23.1% and 42.7%, respectively	To begin with, sampling biases are inherent because Facebook users do not fully represent the entire Australian population, and potential participants who were not Facebook users and/or did not have access to the Internet may have been missed. There was a lack of generalizability. Some of the study's limitations stem from Facebook's own privacy policies. Due to privacy restrictions that prevent Facebook from providing detailed demographic information about users who were exposed to promoted posts, it is not possible to report anything about non-responders when using Facebook. The research team was unable to be seen by the first author due to other Facebook privacy restrictions. The survey costs could not be fully reported, so they were not fully recorded. Finally, exact numbers of participants are impossible to determine. Researchers were unable to obtain any demographic or other information about potential participants who saw an advertisement but did not respond	Over a 10-week period, 1418 participants were successfully recruited using a combination of paid promotions, purposive and snowball recruitment on Facebook, resulting in 810 completed surveys at a low cost per participant. Additional methods for measuring response rates could be beneficial in determining the success of using Facebook posts for recruitment
Chan et al. 2020 [46]	Two themes were found for both registered dietitians (RD) and non-RD blogs: nutrition recommendations and service promotion/sponsorship, several themes were unique to RD or non-RD blogs	A larger sample of blogs could be more representative of the nutrition blogs that are available on the internet. During the data analysis, the researchers were not blinded, but multiple coders were used. The perceptions and retention of blog information were not assessed in this study. In addition, many of the blogs in this sample included images or pictures that could have added to the content but were not included in the analysis	The internet is a powerful tool for disseminating nutrition information to a large number of people. By providing evidence-based nutrition education, addressing current health trends, and making advice accessible to low-income people, RD bloggers can maximise their impact
Gierth et al. 2020 [47]	Prior judgements affect how the following are judged: user comments, the attacked claims, and the claim's source. After adjusting for attitude, people agree more with thematic complexity statements, but only when the comments are made by experts do the comments have a differing impact on perceived argument credibility. Furthermore, comments criticizing researchers' intentions were more effective in lowering perceived credibility, while comments criticizing scientists' expertise had no impact	There are some drawbacks to the research. For instance, the study was largely made up of students. In terms of science reception, students can vary in significant ways from the general population. Nonetheless, the young age of the sample and their willingness to pursue academic studies may have affected their attitudes toward research. We did not provide any conditions under which user comments were missing or irrelevant. We didn't test whether critical user comments affected reputation or trustworthiness, but rather whether discrepancies in content between different types of critical user comments were observed and influenced participants' reasoning. Another drawback is that people's impressions of science-related material on their own Facebook feeds may vary from the screenshots provided to experiment participants	In conclusion, despite the fact that the research was exploratory in nature, it is reasonable to conclude that the content of user comments can influence credibility assessments of science knowledge on social media, and that science communicators should be wary of the complexity argument's comparative effectiveness as an attack on science claims
Kawchuk et al. 2020 [48]	1. Twitter misinformation regarding a spinal manipulation therapy (SMT)/immunity link increased dramatically during the onset of the COVID-19 crisis 2. Activity levels (number of tweets) and engagement scores (likes + retweets) were roughly equal between content promoting or refuting a SMT/immunity link 3. potential reach (audience) of tweets refuting a SMT/immunity link was 3 times higher than those promoting a link 4. majority of tweets promoting a SMT/immunity link were generated in the USA while the majority of refuting tweets originated from Canada	Talkwalker's search results were not compared to those obtained through other services or methods. Twitter is a useful tool for investigating conversations within a social media community, but it is limited in that it does not represent all people on the planet. Some of the data in this study came from proprietary algorithms available from Talker-Walker Quick Search, but we didn't have access to the methods of calculation (e.g. sentiment scores)	During the COVID-19 crisis, there was an uptick in tweets about SMT and immunity. The findings of this study could aid policymakers and others in better understanding the impact of SMT misinformation and devising strategies to mitigate it
Merten et al. 2020 [49]	The majority of pins (91.6%) presented CBD in a positive light, with many citing physical or mental benefits such as relief from anxiety, depression, pain, and inflammation. The majority of pins (98.2%) failed to mention possible side effects or dosage recommendations. User participation was strong in this survey, with 85.2 percent of pins saved and links to commercial sites advertising CBD goods, personal blogs, and social media	This study has several limitations. Since there are so few Pinterest studies, there is a lack of agreement on sampling methodology when performing content analysis (Potter & Levine-Donnerstein, 1999). Pins were only collected for two months, with only every fifth pin being coded, and coding is subject to bias. Furthermore, the degree to which people act on things they pin is unclear on Pinterest, there is no demographic details, and there is no specific way to analyze time range. Despite the study's limitations, it provided insight into the effect of social media on health behaviour and reinforced the need for public health agencies to interact with the general public through social media to educate and counteract harmful health information	Social networking has evolved into a valuable source of health-related knowledge. With only a few credible public health outlets represented, this study revealed widespread approval of the use of CBD products
Allem et al. 2019 [50]	Prevalent topics of posts included using cannabis with mentions of cannabis initiation, processed cannabis products, and health and medical with posts suggesting that cannabis could help with cancer, sleep, pain, anxiety, depression, trauma, and posttraumatic stress disorder. Polysubstance use was a common topic with mentions of cocaine, heroin, ecstasy, lysergic acid diethylamide (LSD), meth, mushrooms, and Xanax along with cannabis. Social bots regularly made health claims about cannabis	Some one-grams and bigrams used to define topics may have multiple meanings that were overlooked in this study. Similarly, it is unclear whether the term "school" always denotes underage use, as college students or other professionals in the educational field may be adult cannabis users. It's possible that the findings do not apply to other social media platforms. The posts in this study were gathered over an eight-month period and may not be applicable to other time frames. The data was collected using Twitter's Streaming Application Programming Interface, which prevented posts from private accounts from being collected. The findings may not apply to all Twitter users or the entire population of the United States. This study was unable to determine the impact of different state cannabis policies on the public's experience with cannabis because not all tweets were covered by the established categories, and topics of conversation were not segmented by geographic location	The findings suggest that processed cannabis products, unsubstantiated health claims about cannabis products, and cannabis use in combination with legal and illegal substances should all be investigated further by public health researchers in the future
Aubrey et al. 2019 [51]	The effect of appearance-framed videos on state self-objectification scores was moderated by age, such that the effect of viewing the appearance-framed videos positively predicted state self-objectification among the younger adolescents. Three topics were chosen for the videos that could either be framed as appearance or health benefits: using sunscreen, drinking water, and doing yoga. In addition, self-objectification mediated the effect of condition on appearance anxiety and on their appearance-enhancing product preferences, again with the predicted effects supported for the younger adolescents in the sample	First, the experimental design of this study allows causal inferences to be drawn about the effects of framing on the outcome variables, however, it is not possible to determine the causal order of the further investigated linkages between self-objectification and appearance anxiety and appearance-enhancing product choice. In addition, in order to address the limitations of the Twenty Statements Test, the measurement of self objectification was done using a novel approach and only preliminary evidence of validity was presented. Furthermore, the study was conducted on a convenience sample of female adolescents, so it is very difficult to extrapolate the findings to other populations, such as females in the same age range who were not enrolled in Qualtrics Panels	Younger adolescent girls were the ones who were most affected by appearance-framed health advice. Furthermore, through self-objectification, the effects of condition on appearance anxiety and appearance-enhancing product choices were indirect. The framing of health advice has an impact on girls' responses to health advice messaging, and the response to the framing of health advice varies by adolescence Younger adolescent girls appear to be the most vulnerable to appearance framing of health advice, as they are likely adapting to pubertal changes and are experiencing chronic monitoring of their appearance for the first time
Cano-Oron 2019 [52]	The sceptical movement occupies a dominant discursive position on Twitter -The perspective is more balanced in digital dailies - 79.1% of the tweets posted using the campaign hashtags were against complementary therapies (CT)s - Tweets defending the use of CTs represented 3.3% of the sample and neutral ones 11.8% - 54.7% of the tweets analyzed did not use or mention sources and the 15.3% that did, usually included links to other messages posted on social networks to websites in favour of CTs - 46.2% of tweets did not refer to the ministry or minister of health accounts - These accounts were mentioned to denounce publicly other accounts relating to these therapies	Sample came from only one campaign of the movement (the sceptical movement) - Only tweets included in the search results of the hashtags were analyzed - Lacked the ability to identify how many people had been exposed to its content	The goal of the study was to determine the sceptical movement’s discourse on complementary therapies in Spain. Hashtags relevant to the campaign, news articles published during the timeline of the study, and interviews with members relevant to the movement were assessed. The results determined that the movement has a strong, dominant position on Twitter in Spain, but is more balanced in digital dailies
Hasan et al. 2019 [53]	1. 65% of subjects had severe irritable bowel syndrome (IBS) pre-treatment, with the remaining 35% having moderate IBS 2. 65% of subjects exhibited a 50-point or more reduction in their total inflammatory bowel syndrome symptom severity (IBS-SSS) score following gut-focused hypnotherapy via Skype, which is regarded as being clinically significant 3. 30% of the subjects exhibited a 150-point or more improvement in their scores 4. Postintervention, 25% of subjects were classified as having severe IBS, 40% moderate IBS, and the remaining 35% mild IBS	A large randomised, controlled, noninferiority trial with more than 100 patients in each group would be required to fully answer this question. However, because Skype treatment is so effective in this desperate group of patients, it would be inappropriate to wait for the results of such a trial, which would be difficult to fund	When the availability of hypnotherapy locally is limited or when subjects find travelling difficult, Skype hypnotherapy appears to be a good alternative to face-to-face treatment
Hu et al. 2019 [54]	The most common adverse events (AE)s and adverse drug reactions (ADR)s in the monitoring system, primarily gastro-intestinal system disorders such as nausea, diarrhoea, and vomiting, were largely similar to those in literature and social media. From social media, 15 AEs were detected (0 unique ADR). When looking at the data, AEs, ADRs, and their affected system-organ classes appeared to be very similar, but they differed in every aspect when details were examined	Since we only collected data for one Chinese patent medicine (CSE), the generalizability of our findings is limited. In China, there is a low level of understanding of active reporting and tracking. CSE safety incidents could be underreported in the ADR tracking database. There were no post-market large-sample, multicenter, well-designed clinical trials for CSE protection. Traditional Chinese medicine items, in particular, need them. In randomized controlled trials (RCT)s, attempting to judge causal inference from AE to ADR was inadequate	The most common AEs and ADRs in our monitoring system, primarily gastro-intestinal system disorders such as nausea, diarrhoea, and vomiting, were largely similar to those in literature and social media. When looking at the specifics, however, data from various sources differed. To gather safety information regarding interventions, various data sources (the surveillance system, literature, and social media) should be incorporated. The distributions of adverse effects and adverse drug reactions from RCTs were the least close to data from other sources
Lacasse et al. 2019 [55]	Fitness (hashtag fitness) was the most cited word (*n* = 5491) - Majority of words were categorized as "good feelings" (*n* = 32,747;51%) and appearance (*n* = 30,351; 42%) - A small amount was categorized as traditional teachings (*n* = 1703; 3%) - Images were mostly of women (*n* = 89; 89%), who were underweight (*n* = 68; 68%), in minimal clothing (70%), demonstrating a basic pose (*n* = 51; 51%), in an indoor environment (*n* = 57; 57%)	Small sample size compared to the large online content	#yoga on Instagram seems to have an emphasis on the physical nature of yoga which falls into line with the commercialization of yoga rather than the traditional teachings of yoga
Lognos et al. 2019 [56]	Patients with breast cancer predominantly use physical (37.6%) and nutritional (31.3%) therapies, according to the findings. Herbal medicine was a commonly mentioned subcategory	Medical characteristics (e.g., type and seriousness of cancer, number of recurrences, treatment time, comorbidities, health status, and risk behaviours) as well as personal (e.g., age), social (e.g., social status), and regional (e.g., France vs Francophonie) information on people who wrote a post are impossible to know. It is hard to say whether the same person shared the same thing several times on various social media sites. The networks' confidentiality laws make it difficult to tell with confidence that all published posts are from cancer patients	The exploratory study of breast cancer patient forums and Facebook discussion groups poses critical concerns about the reliability of complementary and alternative medicine (CAM) information accessible to patients and the role of regulatory authorities in labelling, approval, and surveillance
Majmundar et al. 2019 [57]	User experience (28.90%) and product appearance (21.80%) were predominant themes followed by promotions (10.08%), and flavours (1.01%). About 32.43% of posts referenced cannabis-related solutions, 2.98% of the posts mentioned nicotine-related solutions and 0.11% of the posts mentioned aromatherapy. Average Instagram users (24.89%) posted the majority of posts followed by vape vendors (20.72%), KandyPens’ official account (17.96%), vaping enthusiasts/advocates (10.75%) and influencers (0.45%)	This study's findings may not apply to other social media platforms or time periods. The nature of KandyPens on Twitter and Reddit should be investigated further in the future, as text-based data may provide additional insights. Our findings are specific to KandyPens and may not apply to other companies that sell similar goods. While KandyPens is thought to be popular among teenagers, the demographics of those discussing KandyPens on Instagram were not determined in this study. Additionally, Instagram users making their posts unavailable or deleting them is a characteristic of social media use and may introduce bias in the results if the deleted content is relevant to this research	KandyPens advertises its products as aromatherapy devices, but Instagram posts about the products rarely mentioned their ostensible purpose. To assess implications related to product appeal and abuse liability, future research should consider product design, user experience, and the co-use of nicotine and cannabis with KandyPens
Plachkinova et al. 2019 [58]	The most active contributors to the forums were CAM providers. Some patients also prefer face-to-face encounters with their doctors and complementary and alternative medicine practitioners because they are concerned about the privacy and protection of their health details being shared online. The proposed mobile app. It satisfies the users' requirements and provides them with a user-friendly interface that is intuitive and simple to navigate. People would like to use the service only if they could search for their problem directly, according to three of the six CAM providers. As a result, people should be able to quickly find answers to their questions or diagnoses. There were a total of 4097 users, which means that from March to November 2015, this many people visited the site. Finally, there were 15 organic searches, which indicates that 15 of the visitors arrived from a search engine. Despite the fact that this number is small in comparison to the total number of users, it is higher than anticipated because we did not perform any Search Engine Optimization (SEO). =	The study had a small number of participants which may have influenced the data analysis, particularly given the lack of participation from Western physicians. In the interview sample, as well as among website users, there is an overrepresentation of CAM providers	CAM has been extensively researched, mostly to better understand its advantages and provide proof of effective healing methods. Mobile contact, on the other hand, has mostly been considered for use in health care. It's still unclear how CAM will benefit from Web 2.0 and the development of new communication networks to link patients, CAM practitioners, and traditional physicians. Patients' refusal to share CAM information with their doctors can result in harmful treatments with negative outcomes (Levin, 1996; Plaut, 1995). By involving CAM practitioners in the discussion, the proposed artefact will effectively bridge the divide and restore trust between patients and physicians
Rizvi et al. 2019 [59]	The most common topic group, “use and adverse effects,” accounted for 50 of the 200 topics. The Medical Dictionary for Regulatory Activities = System Organ Classification was used to classify the 15 categories under this subject. The accuracy of identifying questions that correctly correspond with the selected topics was found to be high (90–100% accurate). The findings could help us create a more detailed and organized dietary supplement (DS) resource focused on the information needs of consumers	There were some limitations to this study. We only analyzed questions in the alternative medicine sub-category of the “health” section, so we might have missed dietary supplement mentions in other sub-categories, such as mental health disorders and general health care. To obtain the corresponding questions, we only used preferred DS ingredient names and not their synonyms (e.g., scientific names, common names). Topic modelling has inherent limitations, for example, topics were created based on the statistical word distribution within the questions, resulting in topics with incoherent topic keywords	This study uses correlation explanation (CorEx)-based topic modelling to derive and understand the knowledge needs of consumers around dietary supplements. CorEx-based topic modelling was able to reliably classify specific topics embedded in a wide corpus of Yahoo! Answers data
Webb et al. 2018 [60]	Results revealed that #curvyfit images featured a greater representation of physical appearance–oriented aspects of fitness; #curvyyoga images more often conveyed larger body sizes, shapes, and body-as-process characteristics. Preliminary findings have important implications for counteracting weight-biased perceptions equating thinness with physical fitness and promoting yoga as an important health practice among individuals with a range of body sizes. Our initial stage findings also raise areas of future critical inquiry surrounding the complex messaging at the intersection of fat embodiment, curvy identification, and healthism that are particularly ripe for subsequent qualitative investigation with actual digital media users	Although the sample size was sufficient to detect the majority of effects, it may not have been large enough to capture the presence of lower base rate phenomena. It was difficult to provide more specific information about the distribution of each of those qualities. Directly surveying #curvyyoga and #curvyfit users would be beneficial. The psychological impact of and actual exercise behaviour following exposure to these images for higher-weight individuals who identify as "curvy" or "plus-size" would be beneficial for later stage analyses	The findings support the idea that not all curvy Fitspiration on social media is created equal. On the contrary, despite the fact that both Instagram hashtags included the word "curvy," they tended to reflect philosophically different views of health and fitness. Access to yoga as a practise and challenges to weight-biased stereotypes that dismiss the coexistence of being "fat and fit" increased
Yin et al. 2018 [61]	In total, the 100 videos were viewed more than 36.80 million times. Among them, 52 were consumer videos; 16 were professional videos; and 32 were news videos. Compared to news videos, the odds of consumer videos mentioning what cupping is were 85.90% lower	The findings might not apply to less popular videos or videos in other languages. If videos in other languages were included, the study would be strengthened, especially in areas where cupping therapy is very popular. The study's cross-sectional design failed to capture the changing dynamics of the videos' meta-data (eg, how the number of views changes with time). As cupping becomes more popular, practitioners should be aware of the possibility of misinformation	There are a plethora of consumer videos about cupping therapy on YouTube. The most popular videos on the subject, according to the current study, were derived from news sources. As information from that source becomes more popular among the general public, health professionals will need to increase their presence on YouTube in order to provide reliable information about this alternative therapy, which is becoming more popular
Dai and Hao 2017 [62]	Of all post traumatic stress disorder (PTSD)-related tweets, it was found that 5.3% of tweets were related to marijuana use. Marijuana related tweets reached a large audience. The marijuana related tweets were predominated by supporting opinions and the supporting tweets outnumbered the tweets that were against or neutral about marijuana use	One of the challenges in working with social media data is the amount of “noise” or “chatter” misinformation included in the data. In our study over 10% of marijuana-related tweets were from top 10 users, suggesting that some of these tweets might be sent through power users or twitter bots, not reflecting actual attitudes of the public Study did not assess the trends over time and was unable to establish causal inferences. Used self-reported meta-data that might not have been accurate or not reflective of the users' current location. One of the challenges in working with social media data is the amount of “noise” or “chatter” misinformation included in the data	Twitter data suggest a proliferation of supporting marijuana use for PTSD treatments, especially in the states that legalized medical and/or recreational use of marijuana
Marcon et al. 2017 [63]	Our findings show that there are ongoing debates about the effectiveness and validity of chiropractic. Furthermore, although our research identifies a wide range of claims and debate characteristics, key findings reveal that those advocating “for chiropractic” depend heavily on personal anecdotes while also raising concerns about “pills” and the pharmaceutical industry. Opponents of chiropractic primarily argue that it is not adequately validated by proof or "research," and often provide links to additional literature to back up their arguments. In general, the debates have a low degree of animosity. This study indicates that YouTube is a place where people can explore and debate health-related issues like chiropractic because there are so many different viewpoints being discussed in so many ways. It also illuminates the logic that underpins various chiropractic-related viewpoints and claims	Not specified	This study adds to the body of knowledge about how social media platforms, such as YouTube, become forums for people to express their opinions, reflect on, and discuss health issues (Du, Rachul, Guo, & Caulfield, 2016; Marcon, Klostermann, & Caulfield, 2016; Radzikowski et al., 2016; Vance, Howe, & Dellavalle, 2009). This research focuses on the rhetoric used by both proponents and opponents of chiropractic
Bitcon et al. 2016 [64]	The search revealed a group of bloggers who voiced concerns about the direction Western Herbal Medicine (WHM) is taking and whether they are ethically responsive to global environmental pressures. They share the common value that WHM should remain a health care option that empowers and supports the community, and suggest this is best achieved by maintaining and sharing fundamental skills of plant identification, simple herbal product manufacturing and incorporating both science and tradition in their herbal practice	The study's chosen method may have limited its ability to reach out to other like-minded practitioners. This search did not capture the opinions of bloggers who wrote in languages other than English. Second, the initial sample's keywords were compiled from academic literature, so bloggers who used different terms to describe their blogs would have been overlooked. This bias would have carried over to the second stage of snowball samples, potentially leading to the omission of additional online communities and themes. Furthermore, because Google blog search was the only engine used for the initial collection of blogs, the study may have missed less widely linked blogs	According to Wahlberg (2010), the study demonstrates the existence of a group of herbalists who express their views through nine blogs "with genuine concerns about the direction that herbal medicine is taking." While other herbalists focus on developing professional clinical practise or developing herbal medicine research programmes, this group focuses on promoting herbal medicine as a tool for social justice and a way to strengthen the connection between plants and people
Guo et al. 2016 [65]	Concerned about both the effectiveness of learning and the associated learning load, the team created 20- to 30-min video clips. Fifty pregnant women from a nearby hospital and clinic were enrolled in the mindful yoga programme. A telephone follow-up with these users was conducted to validate their actual use. These participants' comments have also been compiled, which will help researchers better understand their preference and frequency of use. Some of the participants claimed that Facebook was easier to use because it allowed them to practise in the privacy of their own homes. Others said they favoured the digital versatile disc (DVD) because it had a larger screen than their handheld devices, which they used to access Facebook. The majority of the participants alternated between using both methods. One respondent claimed that she learned new yoga poses first by watching a DVD on TV	N/A	Despite the fact that this research is still ongoing, substantial insight in the creation and testing of social multimedia content has been gained. In terms of the Facebook website, it allows users to upload videos that are less than 1024 MB in size. For some users, accessing Facebook via smartphone, Laptop, or Smart TV is a viable choice. The study shows that social media can make health information accessible as the women in the study were able to easily access health educational materials delivered by health care providers through social media sites like Facebook
Krampe et al. 2013 [66]	The biggest surprise resulting from this dance-based therapy Skype session was the enthusiasm of the nursing students. This was the first time many of the students had engaged with older adults in a positive, energetic activity. This process has implications for community and long-term-care clinical settings and could be replicated with minimal resources. The feedback from the TigerPlace older adults varied and included the spectrum of supportive enthusiasm to moderate enjoyment. The challenge to see and hear the Saint Louis University School of Nursing (SLUSON) students posed the biggest problem, given the small screen on the computer used at TigerPlace. Both partners agree that an opportunity awaits SLUSON and TigerPlace to work on specific areas for improvement and enhancing the experience for the older adults: screen size, volume for the older adults, and room size. Another trial will be coordinated in 6 months	The sheer number of students (120) and the presence of five to six older adults was the first hurdle to overcome. The music presented some technical difficulties. The two most important areas at TigerPlace to improve are (1) music facilitation and (2) screen size. TigerPlace could benefit from a larger display. When the music was playing, it was difficult to hear the Lebed Method instructor and nurse at TigerPlace. It would be nice to have a better way to control the volume of the music, such as with a remote. The projector on the ceiling setup in the theatre didn't work	The students were able to see how technology can be used to build interactive relationships with older adults, which was a huge plus. This experience gave a better understanding of the interests and tolerances of the older adult population, as well as hands-on experience with cutting-edge technology in patient care. This technology could be used by nurse educators as well as nurses for activities and tasks that are not cost-effective or practical to complete in person. With the dedicated efforts of a few key individuals, Skype could become a viable option for connecting nurses and nursing students with patients in the future
Marcon et al. 2016 [67]	There were 34 tweets with clear scepticism or criticism of SMT, accounting for 2.68 percent of the overall sample (n = 1267). As a result, 2.68 percent of tweets in the total corpus (95 percent confidence interval 0–6.58 percent) express explicitly sceptical or critical views of SMT. There are also several tweets highlighting the health benefits of SMT for health problems like attention deficit hyperactivity disorder (ADHD), the immune system, and blood pressure, which receive little critical attention. There are a few tweets in the corpus that highlight the dangers of "stroke" and "vertebral artery dissection" (0.1 percent)	There are a few limitations to this study that should be considered. It can be difficult to compile a comprehensive set of tweets on any subject due to the nature of Twitter conversations and the very restricted access given by Twitter's application programming interfaces (API). Also, other possible words like "chiro" and "spinal change" are also present on Twitter, which may result in datasets with slightly different outcomes. Finally, considering the fact that December 2015 was selected at random, there is no reason to assume that other time periods will be slightly identical or dissimilar. Despite these limitations, this study shows how risk discussions and critical views on efficacy are almost non-existent on Twitter	There is a lack of skepticism surrounding the effectiveness of SMT on Twitter as the majority of tweets substantiated and encouraged chiropractic and SMT as sound health practices. While some critical voices of SMT are gaining traction, questions remain about how widely this knowledge is being disseminated
Robertson 2016 [68]	Treatment group A had substantial results between the mid/post-test and pre/post-test, but not treatment group B, indicating that a stimulation technique applied during the first two weeks encouraged them to visit the blog site even during the nonstimulus situation the last two weeks. Both groups gave the blog high scores for its utility in collecting knowledge about neonatal intensive care unit (NICU) music therapy services	Not specified	Overall, the blog seemed to increase primary care providers' understanding of NICU music therapy services. It was a successful way to disseminate relevant intervention knowledge as well as new research findings that are critical to the success of music therapy in the NICU. Participants were able to easily access valuable educational knowledge at their leisure, alleviating time constraints that are common in the medical community as a primary care provider
Keim-Malpass et al. 2013 [69]	The following thematic classifications were described: awakening, new identities (that incorporate loss), the good stuff, and release. The desire to achieve strength, peace, and the ability to heal both physically and emotionally were common attributes expressed before, during, and after CAM use. All of the women who utilized CAM described a similar series of events that culminated in a loss of control of what was happening to their bodies, usually acting as a point of initiation for complementary modalities. As treatment commenced, many of the young women were faced with new bodily identities that encompassed both physical changes and limitations in previous activities. The CAM use was often initiated by high symptom burden and an empowering desire to take a proactive and positive complementary approach to treatment. The described CAM use had both short-term impacts for combating negative disease experiences as well as longer-term impacts for enhanced cancer survivorship and positive lifestyle changes through stress reduction, mindfulness, physical activity, and more balanced nutritional choices	Beyond the experiences presented, the data is not generalizable. The identity of the person was not recorded. As a result, medical records could not confirm any disease or treatment-related details. Furthermore, because of the naturalistic approach, topics were only written about and discussed if they were important to the blog author	Online illness blogs allow researchers to gain a better understanding of the patient's entire experience through personal accounts, and they contribute significantly to the body of knowledge surrounding cancer in young adults and the use of complementary therapies
Krampe et al. 2016 [70]	1. The visual experience was better for the front and second rows. 2. All of the older adults (100%) reported hearing the audio adequately, regardless of position in the room. 100% of older adults with student partners enjoyed seeing the dancers along with the older adults in the second row. 3. The older adults with student partners and the second row reported 100% satisfaction and willingness to join this program again. 4. 100% of older adults reported hearing the audio adequately	There was some delay in audio with video, which may have been because Fuze was streaming both the video and the uploaded audio content at the same time. The visual component of Fuze was problematic at the school of nursing (SON). Participants at the SON stated that the video quality was not always sharp but somewhat blurry. This may be attributed to the fact that it was being streamed via Wi-Fi at the partner facility. There did not appear to be a wired Internet connection available near the TV at the facility. This issue might have been resolved with a wired connection. One important factor for improvement will be to avoid overwhelming the older adults. “It is difficult to watch the screen and dance leader at the same time” was a comment from 1 of older adults in the front row who had a student partner. The dance leader was stationed outside the camera view; a position in front of the large screen TV, off to one side, may improve this problem	Overall, Fuze is a feasible, engaging, and satisfying approach for dance-based therapy, with better audio and visual performance than Skype. The use of synchronous technology to provide therapeutic activities for older adults is an area of research and exploration that appears to have great potential. More trials are needed
Walden 2013 [71]	Individual and group credibility can be projected to stakeholders via blogs	There are a few shortcomings of this study. Interviewees may have been unable to go into great depth due to the short duration of the interviews. Though interviewees were wary of outside scrutiny, more in-depth probing about how naturopathic physicians perceive issues in their field may have been helpful. Also, these are my findings based on this small sample of 18 people, limiting generalizability	Blogs are recent examples of grey or alternative health literature that act as a “represent all of us” community feature. This study serves as a springboard for more qualitative and quantitative research in these fields, especially in the holistic medical community, to better understand how health information can be disseminated and medical practitioner identity can be discussed on Web 2.0 technologies
Gregory et al. 2012 [72]	Although very few educational institutions listed by the American Music Therapy Association provided documentaries on their websites, YouTube documentaries by a variety of posters were very prevalent. The largest proportion of YouTube documentary postings were originally created by professional news organizations and were, not surprisingly, effective in conveying objectives within videos of adequate length and audio/visual quality. Content included both video footage of clinical interactions showing clients and music therapists and didactic information through narrative overlays, interviews, and brief talks. However, professional credentials of music therapists were provided less frequently than credentials of non-music therapy interviewees and therapists, which highlighted missed opportunities for informing viewers of professional designations for music therapists. In addition, music therapy orientations were not identified in more than half of the documentaries, which prevented viewers from learning that diverse approaches exist within the music therapy profession. Even with these limitations, comparisons with results from a similar examination of YouTube music therapy session videos suggest that, generally speaking, online music therapy documentaries probably provide a more effective and accurate format for current educational and outreach purposes	Music therapists' professional credentials were provided less frequently than those of non-music therapy interviewees and therapists, highlighting missed opportunities to inform viewers about music therapists' professional designations. Furthermore, music therapy orientations were not identified in more than half of the documentaries, preventing viewers from learning about the variety of approaches available in the field of music therapy	Despite these challenges, comparisons to results from a similar study of YouTube music therapy session videos suggest that, in general, online music therapy documentaries are a more effective and accurate format for current educational and outreach purposes

## Findings from thematic analysis

Three main themes were identified through our thematic analysis. These themes are described in the paragraphs below. Sample excerpts from included studies representative of each of these themes are shown in Table 4.

**Table 4:** Quotes from Eligible Studies Supporting Themes

First Author and Year	Theme 1: Positives & Negatives	Theme 2: Misinformation	Theme 3: Challenges
Barnes et al. 2020 [45]	N/A	N/A	“…sampling biases are inherent as Facebook users are not fully representative of the entire Australian population, and potential participants may have been missed if they were not Facebook users, and/or did not have access to the Internet. For the current study, although target numbers were met for breastfeeding participants, and nearly met for pregnant participants, it is not possible to generalise the results to the wider Australian pregnant or breastfeeding population. […] Some limitations to the study also arise from the privacy standards of Facebook itself. It is not possible to report anything about non- responders when using Facebook due to the privacy restrictions that prevent Facebook from giving detailed demographic information about users who were exposed to promoted posts. […] Due to other Facebook privacy restrictions, shares to, or within, closed or secret (private) Facebook groups, or as private messages from outside the research team were not able to be viewed by the first author. […] Finally, it is not possible to determine the exact numbers of participants who found out about the survey on Facebook and viewed the initial survey information page but did not then click through to the actual survey.”
Kawchuk et al. 2020 [48]	N/A	“Twitter misinformation regarding a SMT/immunity link increased dramatically during the onset of the COVID crisis.”	“Although Twitter provides a window into conversations within a social media community, it is limited in that it does not represent all persons in the world. Presently, Twitter ranks 13th in total monthly users; Facebook has 2.45 billion active monthly users compared to Twitter’s 340 million.”
Merten et al. 2020 [49]	Positive: “The majority of pins positively portrayed CBD 91.6% (n1⁄4207) whereas 8.4% (n1⁄419) were balanced, and no pins negatively portrayed CBD.”	“The majority (91.6%) of pins positively portrayed CBD with many claiming a physical or mental benefit including anxiety, depression, pain, and inflammation relief. Most pins did not (98.2%) address potential side effects or recommend dosage…This study revealed widespread acceptance of the use of CBD products with minimal information from reliable public health sources represented.”	“Similar to Twitter and Facebook, Pinterest pins are searchable in search engines such as Google and Yahoo! unless the user has adjusted their privacy settings to add a secret board […] with Pinterest, it is unknown the extent to which people act upon items they pin, there is no demographic information, and there is no precise way to analyze time range.”
Allem et al. 2019 [50]	Positive: “Among health and medical, cannabis was suggested to help with cancer, plantar fasciitis, Crohn’s disease, sleep, pain, anxiety, depression, trauma, and post- traumatic stress disorder, among others.”	N/A	“…findings may not extend to other social media platforms. The posts in this study were collected from an 8-month period and may not extend to other time periods. Data collection relied on Twitter’s Streaming Application Programming Interface, which prevented collection of posts from private accounts. Findings may not generalize to all Twitter users or to the US population. Not all tweets were covered by the established categories, and topics of conversation were not segmented by geographic location, preventing this study from determining the impact of different state cannabis policies on the public’s experience with cannabis.”
Cano-Orón 2019 [52]	Negative: “The majority (79.1%) of the tweets posted using the campaign hashtags were against CTs. Whereas tweets defending the use of CTs represented 3.3% of the sample and neutral ones 11.8%.”	N/A	N/A
Hu et al. 2019 [54]	Negative: “In monitoring system, there were 610 AEs reports associated with CSE, in which 537 (88.03%) were suspected ADRs (10.49% certain). […] In the literature, 5568 AEs were identified, of which 86 (1.54%) were classified as ADRs (1.54% certain). 271 AEs were identified from 108 RCTs (n = 4682). ADR rate in RCTs was 0.021%. Baidu post bar (351 themes) and Sina micro-blogs (309 posts) published a total of 660 posts. We found no useful safety information from Baidu post bar, while we identified from Sina micro-blogs 15 AEs (unassessible/unclassifiable) in which none could be judged as certain or likely ADRs due to vague descriptions.”	N/A	“Our data was only for one Chinese patent medicine (CSE), thus the generalizability of our results is limited.”
Lacasse et al. 2019 [55]	Positive: “The most common words associated with #yoga included #fitness, #gym, #workout, and #fit, suggesting that the online depiction of #yoga is based around the physical benefits (e.g., being thin/fit from *asana*) as opposed to the more in‐depth limbs of yoga, such as meditation or *dhyana* that leads one to experience yogic enlightenment. According to the CPM, both the author/sender and the audience/receiver may be perpetuating the message of physical benefits based on the most commonly used words (as opposed to spiritual enlightenment), thus supporting the CMC theory of using Instagram as a valuable source of communication that may be changing one’s beliefs and attitudes toward the practice of yoga.” Positive: “Moreover, popular/emerging themes/text around #yoga suggested that good feelings and appearance were the largest content categories. […] Further, the current data support previous work that practicing was associated with the societal values of reducing stress and feeling positive as well as increasing beauty and muscularity.”	N/A	N/A
Lognos et al. 2019 [56]	N/A	“The study underlines the power of digital social networks to share—disseminate—recommend practices across borders of which health professionals may have little awareness. Some patients become precursors, *beta testers*, of solutions never proven or whose manufacturing quality remains to be verified. The study raises important questions about the reliability of CAM information available to patients and regulatory authorities’ responsibility for labeling, approval, and surveillance. The results sensitize health professionals and authorities to the power of forums and discussion groups to make known beneficial but also potentially dangerous solutions that currently escape the purview of regulatory and monitoring systems.”	“Given the confidentiality required for the use of the social network data studied and the ethical framework of this study, it was impossible to know the medical characteristics (eg, type and severity of cancer, number of recurrences, treatment period, comorbidities, condition health, and risk behaviors) or personal (eg, age), social (eg, social status), and geographical (eg, France vs Francophonie) information on people who wrote a post. Moreover, it was impossible to know if posts were repeated several times by the same person, including on different social networks. Finally, the rules of confidentiality of the networks do not make it possible to affirm with certainty that all published posts emanate from patients with cancer. For example, companies can use these tools by creating virtual patients to promote their nonpharmacological products. Relatives of a sick person can also register to search for information. Impostors could also be spreading false medical information.”
Majmundar et al. 2019 [57]	Positive: “Themes highlighting User experience (28.90%) and Product appearance (21.80%) were the most predominant followed by posts classified as promotions (10.08%) and those highlighting flavours (1.01%) […] majority of the posts were posted by average Instagram users (24.89%) and vape vendors (20.72%) followed by KandyPens’ official account (17.96%), vaping enthusiasts/advocates (10.75%) and influencers (0.45%).” The authors define “vaping enthusiasts/advocates” as when “the account name mentions vaping or cannabis-related terms but does not sell products” and define “influencers” as when “the account promotes KandyPens by explicitly stating it using specific hashtags such as ‘#ad’ ‘#sponsored’.”	N/A	N/A
Rizvi et al. 2019 [59]	Positive & Negative: “The top three groups with the most number of respective assigned categories and topics, which can be regarded as the information most sought by consumers, are: “use and adverse effects”, “product-related”, and “healthy life style” […] Extracted information pertaining to any symptom or sign could either be an indication or an adverse event of a DS, (e.g., diarrhea, abdominal pain, palpitations, headaches); therefore, uses and adverse effects were combined as one group, “use and adverse effects”. We found a higher number of topics and the associated number of questions concerning: gastrointestinal system (specifically diarrhea and constipation); psychiatric (mainly anxiety and depression); and skin and subcutaneous tissues (primarily acne and UV protection). We also had a “mixed group”, having keywords corresponding to more than one system. For “product-related groups”, we merged categories like dose, dose from, preparation because of their co-occurrence under one topic (e.g., Topic #43). Under the “healthy life style” group, the topics were mostly around eating healthy and weight control/exercise.”	N/A	“The purpose of this research study is to understand the information needs of DS consumers by analyzing questions coming directly from consumers and in their own language. The goal is achieved by using Correlation Explanation (CorEx)—a topic modeling algorithm on the title and body of each question under the Q&A section of the Yahoo! Answers database in order to unveil the “topics” around DS information needs. We generated a list of coherent topics that more accurately represent the areas of DS-related information and associated DS ingredients that consumers are most interested in. We will also evaluate the accuracy of the CorEx method in correctly identifying the topics from social media. In the future, the knowledge gained from this study could be used as a guide for developing more meaningful DS resources for consumers that are better aligned with their information needs.” “This information provides essential knowledge on the use of DS for various specific reasons and needs further exploration. […] We analyzed only questions belonging to alternative medicine sub-category under “health” section and might have missed dietary supplement occurrences under other sub-categories, e.g., mental health conditions, general health care. […] There are inherent limitations to topic modeling e.g., topics were generated based on the statistical word distribution within the questions and thus topics with incoherent topic keywords were also generated.”
Yin et al. 2018 [61]	N/A	“In total, the 100 videos were viewed more than 36.80 million times. Among them, 52 were consumer videos; 16 were professional videos; and 32 were news videos… Health professionals could engage more with YouTube by providing clear and authentic information about a popular alternative therapy.”	“The research team focused the study on the 100 most widely viewed, English-language, YouTube videos on cupping, and the results may not be generalizable to the less popular videos and videos in other languages. The study would be strengthened if videos in other languages were included, particularly in places where cupping therapy is highly popular. The cross-sectional design of the study did not capture the changing dynamics of the meta-data of the videos (eg, how the number of views changes with time).”
Dai and Hao 2017 [62]	Positive: “The marijuana related tweets were predominated by supporting opinions and the 213 supporting tweets outnumbered the tweets that were against or neutral about marijuana use by a 214 ratio of 8.6 to 1. The tweets that support marijuana use had a higher number of tweets per user 215 (2.6 vs. 1.8, P < 0.001) and a higher number of followers per user (19,495 vs. 10,467, P < 0.001) 216 than those against or neutral about marijuana use.”	“One of the challenges in working with social media data is the amount of “noise” or “chatter” misinformation included in the data. In our study over 10% of marijuana-related tweets were from top 10 users, suggesting that some of these tweets might be sent through power users or twitter bots (Benevenuto et al., 2010), not reflecting actual attitudes of the public.”	“…our study is a cross-sectional in examining PTSD related tweets over 258 days. We did not assess the trends over time and were unable to establish causal inferences. […] Our research is based on a sample of tweets extracted with selected search keywords related to PTSD and marijuana use. […] Given the sample biases inherent in the Twitter data, the findings from this study should be interpreted with caution and they may be more reflective of perceptions for a certain population. […] Fourth, one of the challenges in working with social media data is the amount of “noise” or “chatter” misinformation included in the data. In our study over 10% of marijuana-related tweets were from top 10 users, suggesting that some of these tweets might be sent through power users or twitter bots, not reflecting actual attitudes of the public.”
Marcon et al. 2017 [63]	Negative: “The “against chiro” group labelled chiropractic and chiropractors with five different negative terms: bullshit/bs; snake oil salesman; witchcraft (voodoo, etc.); a con/scam (taking advantage of gullible people); quacks (quack, quackery, quackropractic); and hacks. This group also used four more elaborate arguments that included: (1) equating chiropractic with the placebo effect; (2) delegitimizing the practice by stating that it is not supported by science, evidence, or evidence-based science; (3) stating explicitly that chiropractic does not cure anybody, thus requiring continual treatment, and (4) suggesting that chiropractic can be risky or dangerous. In addition, commenters in this group (1) provided links to studies/articles to support arguments and (2) expressed nuance, stating there are “good chiropractors” or that chiropractic can be effective in some situations, but that numerous medical benefits should not be attributed to the treatment.” Positive: “In the “for chiro” group, argumentative characteristics fall broadly into two overarching categories: (1) expounding the benefits of chiropractic and (2) raising critical issues in medical care which chiropractic avoids. Regarding benefits, commenters talk about chiropractic being natural and using natural processes; getting to roots of a problem (not treating merely symptoms but underlying is- sues); helping with migraines; being safe; having a long history (“over 4,000 years”), and being science-based. In addition, personal anecdotes are used to make claims of chiropractic efficacy.”	N/A	N/A
Bitcon et al. 2016 [64]	N/A	N/A	“Views of bloggers writing in languages other than English were not captured in this search. Secondly, the keywords used for the initial sample were compiled from existing academic literature therefore bloggers that used different terms to describe their blog would have been missed. This bias would have then carried through to the second stage of snowball samples and potentially resulted in missing other online communities and themes. In addition, the research may have missed less widely linked blogs as Google blog search was the only engine used for the initial collection of blogs.”
Marcon et al. 2016 [67]	Negative: “Of all tweets analyzed in Corpora 1 and 2 (*n* = 1200), a total of 77 tweets (6.42%), 95% CI (2.52%-10.32%) contained skeptical or critical sentiment. Following in-depth analysis, 25 of the 77 tweets contained explicitly skeptical or critical content, representing 2.08% of the more general Twitter discourse, 95% CI (0%-5.98%). In Corpus 3: “spinal manipulation” (*n* = 67), 25 tweets, 37% of the corpus, contained skeptical or critical sentiments. Following in-depth analysis, 9 of the 25 tweets contained explicitly skeptical or critical content, representing 13% of the Corpus.”	“In the abundance of tweets substantiating and promoting chiropractic and SMT as sound health practices and valuable business endeavors, the debates surrounding the efficacy and risks of SMT on Twitter are almost completely absent. Although there are some critical voices of SMT proving to be influential, issues persist regarding how widely this information is being disseminated.”	N/A
Keim-Malpass et al. 2013 [69]	Positive: “Through CAM use, and yoga in particular, an awakening was described, where the participants were able to reconnect with their bodies in a more meaningful way Many of the young women were acutely aware of their bodily experiences throughout the cancer trajectory. The young women became “in tune” with minute sensations and changes that they were not aware of prior to the cancer diagnosis. Such descriptions were found in several narratives and were often recounted as a positive encounter that coincided with the CAM experience. […] This awakening and reconnection through CAM use was uniformly characterized as a positive sentiment. For bodies that were somewhat alienated as “not fully their own” during the cancer journey, use of CAM represented an option where young women could learn to trust their bodies again.” Positive: “… suggests that many found strength within CAM modalities (particularly yoga) to help reshape and focus on the evolving identity post-diagnosis.” Positive: “For many of the young women, using CAM signified the women taking positive directions to control their health. The descriptions of “the good stuff” embedded within their narratives represented food and nutrition modalities used to make changes in nutritional choices, support the immune system, and help alleviate side effects from treatment.” Positive: “One of the most powerful elements of CAM was the sense of release that would accompany yoga, acupuncture, meditation, or guided imagery. The release that occurred allowed the women to transcend their physical body and be present in the moment. […] The descriptions and use of CAM by the young women highlights the moments of solace and reconnection to themselves during periods of cancer treatment. The CAM use was often initiated by a high symptom burden and an empowering desire to take a proactive and positive complementary approach to treatment. The described CAM use had both short-term impacts for combating negative disease experiences as well as longer term impacts for enhanced cancer survivorship and positive lifestyle changes through stress reduction, mindfulness, physical activity, and more balanced nutritional choices.”	N/A	“Because qualitative methodology was used, the data lack generalizability beyond the experiences presented. In addition, because the participants were only accessed through online public websites, identity was not captured. Therefore, no disease or treatment-related details could be confirmed by a medical record.”
Walden 2013 [71]	N/A	N/A	“I do not wish to overstate the functional appeal of blogs. As discussed earlier, a search for naturopathic blogs revealed several dozen blogs that appeared at the top of search engine results but were apparently abandoned. Three bloggers who were originally interviewed for this study stopped blogging at their sites in 2010, while another three interviewees had published five or fewer posts through November 2011. Such limited activity in nearly a third of this study’s sample reveals the challenge of regularly blogging. Most interviewees, regardless of the number of entries they write, observed that blogging takes a lot of time. There are pressures to get it right and to “represent all of us” in an accurate and science-based manner. To spread positive information about the discipline, naturopathic physicians appear to start blogs with great enthusiasm yet blogging interest may wane after a certain point.”

### Theme 1: To share user beliefs, attitudes, and experiences about CAM

Several studies provided insight into the beliefs, attitudes, and experiences of CAM users [49, 50, 52, 54, 55, 57, 59, 62, 63, 67, 69]. Three subthemes developed among the studies: negative beliefs and attitudes about CAM use, positive beliefs and attitudes about CAM use, and positive and negative experiences of using CAM. Studies included in this theme described a range of beliefs, attitudes and/or experiences related to CAM which were coded into categories based on whether they reflected predominantly positive or negative views.

#### Subtheme 1.1: Negative beliefs and attitudes about CAM use

The first of the three subthemes found among the studies was negative beliefs and attitudes about CAM use. A number of studies identified negative beliefs and attitudes about CAM treatments that were posted on social media [52, 54, 63, 67]. One study sought to “analyze the sceptical movement’s discourse on complementary therapies in Spain, as well as comprehend its mobilisation against these therapies” [52]. The authors reviewed more than 6000 posted tweets and found that 79.1% were against or not in favour of CAM treatments. The common themes conveying concerns about CAM among the tweets were “anti-science”, “fighting against harmful, for-profit practices”, and protecting “the most vulnerable [who have] little knowledge of science”. In a different study, researchers investigated the presence of critiques and debates surrounding the effectiveness and risk of chiropractic and spinal manipulation therapy (SMT) on Twitter [67]. It was found that the efficacy of these CAM treatments was rarely questioned or doubted. Additionally, the potential risks were rarely mentioned or debated. However, of the few tweets that were skeptical or critical about the use of chiropractic and SMT, most had been liked and retweeted, demonstrating that many skeptical or critical perspectives of CAM use elicited high engagement among social media users even though their voices were marginal in number.

#### Subtheme 1.2: Positive beliefs and attitudes about CAM use

Three studies intended to analyze the public beliefs and attitudes expressed about CAM use on social media and assess whether they were predominantly in favour of or against CAM use [49, 62, 69]. One study analyzed descriptions of CAM treatments used by young women diagnosed with cancer who kept an online cancer blog [69]. The descriptions of CAM treatments were uniformly expressed in a positive and empowering manner by the young women. Additionally, two studies assessed how cannabidiol (CBD) products were presented on popular social media platforms, including Twitter and Pinterest [49, 62]. Both studies found that the majority of posts presented CBD in a positive light, with many citing physical or mental benefits, such as relief from anxiety, depression, pain, and inflammation. Similarly, a study investigating posts on Instagram related to yoga found that most posts emphasized the physical benefits of yoga and used words like “fitness” when describing yoga [55]. Another study that focused on cannabis-related conversations on Twitter discovered that the topics of conversation ranged from using cannabis for the first time to the legality and therapeutic value of cannabis [50]. Regarding the therapeutic value, posts discussed numerous medical conditions such as Crohn’s disease, cancer, post-traumatic stress disorder, anxiety, and depression that are being treated or have the potential to be treated by cannabis.

#### Subtheme 1.3: Positive and negative experiences of using CAM

Four studies found that the information most sought by consumers on social media sites was relating to the experiences of past users of CAM treatments [57, 59, 63, 69]. For example, one study analyzed questions posted on Yahoo! Answers relating to dietary supplement ingredients under the subsection, “Alternative medicine” under the section, “Health” [59]. It was found that the information most sought by consumers, defined by the greatest number of posts, was relating to the uses and adverse effects of dietary supplements. The most common uses of the dietary supplements were respiratory, thoracic & mediastinal disorders, cardiovascular & lymphatic system disorders, and psychiatric disorders, while the most common adverse effects were diarrhea, abdominal pain, palpitations, and headaches. Another study examined descriptions of CAM use among women diagnosed with cancer who maintained an online cancer blog [69]. The study found that the women used CAM treatments for a multitude of reasons, including the feeling of a loss of control, negative symptom experiences, as a means of reconnection to their bodies, and because of the desire to have a more active engagement in their medical care. Another study investigated social media as a platform to share information about the safety of Chinese patent medicine [54]. The authors found that there were a substantial number of posts on online blogging platforms about individuals experiencing adverse effects while using Chinese patent medicine.

A different study analyzed posts on Instagram related to KandyPens, an e-cigarette company that markets its products as aromatherapy devices [57]. The most predominant themes displayed in the posts were user experience and product appearance. Additionally, one study found that individuals had both negative and positive experiences with a popular CAM treatment, chiropractic [63]. The study explored debates surrounding chiropractic in the comment section of popular chiropractic-related videos on YouTube. The comments section was split between individuals with negative and positive beliefs, attitudes, or experiences regarding chiropractic. Individuals who held negative beliefs about CAM tended to argue that therapies such as chiropractic were not supported by sufficient evidence or “science”. Individuals who held positive beliefs about CAM usually alluded to personal experiences and raised issues with conventional medicine and the pharmaceutical industry.

### Theme 2: Misinformation about CAM on social media

Misinformation about CAM being shared on social media was another theme that emerged. We did not make a judgement on what is considered misinformation. Instead, whether something was deemed misinformation was determined and stated by the authors of the included studies themselves. Numerous studies discussed how social media acts as a vehicle for the spread of misinformation about CAM [48, 49, 56, 61, 62, 67]. For example, since the onset of the COVID-19 pandemic, the quantity and popularity of tweets suggesting a link between spinal manipulation therapy (SMT) and immunity increased substantially [48]. Furthermore, posts about CAM on breast cancer patient social forums and Facebook groups have raised critical concerns about the reliability of information accessible to patients [56]. For example, it was found that some patients test CAM therapies that have not been shown to be safe nor effective or whose manufacturing quality have not been verified [56]. Additionally, information that is potentially dangerous can be shared on social media and without being reviewed by regulatory and monitoring systems [56]. However, studies suggest that not all information about CAM on social media, whether factual or inaccurate, is equally trusted by social media users [47]. For example, for naturopathic physicians, citing research articles in their blogs has been suggested as a valuable tool to build credibility both for them individually and for their discipline as a whole [71]. Additionally, one study's researchers showed their participants Facebook posts about research which found that homeopathy leads to health risks [47]. This study found that if comments criticize the intentions of the researchers rather than their expertise, they are more likely to effectively reduce perceived credibility of these Facebook posts [47]. Various studies found that there is a lack of qualified voices represented in social media posts about CAM [49, 61, 67]. For example, out of the 100 most widely viewed YouTube videos on cupping therapy, only 16 were created by qualified professionals [61]. Studies also stated that the high prevalence of misinformation about CAM on social media can help policymakers better understand and devise strategies to mitigate it, and raises questions about regulatory authorities’ role in labelling, approval, and surveillance [48, 56].

### Theme 3: Challenges with social media research in the context of CAM

More than a third of studies identified challenges with social media research in the context of CAM [45, 48–50, 54, 56, 59, 61, 62, 64, 69, 71]. There were three subthemes that emerged across these studies, each representing a specific challenge with performing high-quality social media research in the context of CAM including: the inherent sampling biases, the privacy standards of social media platforms, and the difficulty identifying posts that represent the actual attitudes of the public. These subthemes highlight the difficulty in collecting a representative sample in social media research in the context of CAM. Although studies utilized different definitions of CAM and surveyed distinct CAM treatments on social media, all made specific determinations as to where to draw their search criteria [45, 48–50, 54, 56, 59, 61, 62, 64, 69, 71]. Studies with a narrow search criteria within a subset of CAM did not necessarily have a small sample size, therefore having a narrow search criteria was not viewed as a challenge with social media research in the context of CAM. Studies included in this theme reported a range of challenges related to social media research in the context of CAM.

#### Subtheme 3.1: Sampling biases are inherent

More than a third of studies reported that a challenge with social media research in the context of CAM was that sampling biases are inherent because social media users are not representative of the general population [45, 48, 50, 54, 59, 61, 62, 64, 69, 71]. Three studies included a sample of social media users from a single platform (e.g., Twitter) and attempted to draw conclusions about the broader population beyond those who engaged in social media [45, 50, 59]. While the authors of these studies were able to collect a sufficient sample of social media data, they acknowledged that obtaining a representative sample of the general population was difficult because individuals who chose to post to a particular social media platform may not be typical of the general public and their online activity may not reflect their behaviour in other settings [45, 50, 59]. Two studies that analyzed activity on Twitter related to particular types of CAM use also mentioned that their findings were not generalizable to the broader population and that they may have missed potential participants that had private accounts or did not have access to the internet [48, 62]. Additionally, two studies that utilized qualitative methodology to analyze activity on online blogs recognized that their data was not generalizable to the general public [64, 69]. The two studies also noted that participants were only accessed through online blogs so their identities were not captured, and thus, no medical condition or treatment-related details could be confirmed by medical record. Furthermore, various studies focused on posts from a single social media platform (Twitter) and acknowledged that their findings may not extend to other social media platforms [45, 48, 50, 69, 71]. Furthermore, two studies that only collected data on a single CAM treatment (e.g., Chinese patent medicine) on social media recognized that their findings may not extend to other CAM treatments [54, 59]. Two studies also acknowledged that the views of social media users who posted in languages other than English were not captured [61, 64]. It is important to note that for some studies, surveying a representative sample of the general population was not part of the study design, but rather to obtain an adequate sample of individuals who were current users of a specific type of CAM (e.g., spinal manipulative therapy) [41, 48, 49, 51, 56, 58].

#### Subtheme 3.2: Privacy standards of social media platforms

Furthermore, some studies mentioned that the reason there were challenges with social media research was because of the rigid privacy restrictions that prevented collecting detailed demographic information about users who were exposed to or interacted with a post on social media, but chose not to respond [45, 49, 56]. Authors of three studies, which explored either Facebook or Pinterest, discussed this challenge in their research [45, 49, 56]. For example, one study's researchers analyzed the use of Facebook to recruit a target group of people to a survey on a CAM product [45]. The study discussed its recruitment method, which was primarily through Facebook advertisements, and the challenge of having a limited ability to assess the magnitude of any differential response bias because so little is known about nonrespondents (i.e., those who viewed the study recruitment advertisement, but did not click on it). Similarly, another study discussed the difficulty with conducting social media research because social media platforms such as Pinterest do not share demographic information, the time of activity, or the extent to which users act upon the items they pin [49].

#### Subtheme 3.3: Challenges with identifying posts that represent the actual attitudes of the public

Some studies described that one of the challenges of working with social media data was identifying posts that represent the actual attitudes of the public [61, 62]. One study analyzed the public attitudes towards medicinal cannabis use for PTSD on Twitter [62]. The study reported that over 10% of all marijuana-related tweets were posted by the top 10 most popular cannabis-related Twitter accounts. This suggests that some of the tweets included in the study may have been sent through power users or Twitter bots [62, 73]. One study analyzed user-generated content found on YouTube about the practice of cupping therapy as a form of pain management [61]. The authors focused the study on the 100 most widely viewed English-language YouTube videos on cupping and noted that the results may not be generalizable to less popular YouTube videos.

## Discussion

The purpose of our scoping review was to provide a summary of the research on the ways in which social media is used in the context of CAM. This study identified 29 eligible articles which were published between 2012 and 2020. The amount of available literature on this topic, while not overly voluminous, presents a broad range of social media platforms analyzing a variety of CAM treatments such as chiropractic, yoga, Chinese patent medicine, and medicinal cannabis. To our knowledge, this is the first study to perform a systematic search of the peer-reviewed and grey literature on this topic. As CAM-related health therapies and products are highly mediatized with a strong presence on social media platforms which can influence individual’s health beliefs, attitudes, and subsequent behaviours, the present study's findings may be of value to both health care practitioners and researchers alike.

### Resources for practitioners, researchers, and patients: abundant, but of unclear quality

This scoping review also provides readers with the list of eligible articles included in the present study which may aid in their understanding of how CAM is portrayed in social media. While the eligible articles that were included in this scoping review have been developed and evaluated to some degree by academic researchers, the present study was only designed to scope out the number of CAM-related social media studies and their key characteristics. As expected, most eligible studies analyzed well-known social media platforms such as Instagram [55, 57, 60] and Twitter [50, 62, 67], however, some others examined lesser-known social media platforms including online illness blogs [69] and patient forums [56]. Furthermore, 12 eligible articles lacked generalizability due to challenges with conducting social media research including the inherent sampling biases [45, 48, 50, 54, 59, 61, 62, 64, 69, 71], the rigid privacy standards of social media platforms [45, 49, 56], and the difficulty identifying posts that represent the actual attitudes of the public [61, 62]. In addition, most studies analyzed data about a single type of CAM treatment (e.g., chiropractic) instead of multiple types of CAM treatments, which may have resulted in a lack of generalizability of study findings to other social media platforms and/or other CAM treatments.

### Comparative literature

#### Discussion of Theme 1: to share user beliefs, attitudes, and experiences about CAM

With regard to comparative literature pertaining to the use of social media to share user beliefs, attitudes, and experiences about CAM therapies, several studies reported that social media can be a useful tool for patients, physicians, and other health care professionals because it pools information on patients’ evaluations of, and health outcomes from CAM therapies [43, 63, 64]. For example, one study explored the interest of patients with breast cancer in CAM-related social media posts [43]. The study indicated that patients during and after treatments for breast cancer had a strong interest in social media posts about CAM interventions to complement their approved treatments. Another study found that 8% of cancer related information shared on Facebook was about CAM therapies [63]. Moreover, one study found that social media has been used to discuss CAM related therapies for glaucoma, with 40% of glaucoma related tweets associated with CAM therapies [64].

#### Discussion of theme 2: Misinformation about CAM on SOCIAL Media

A number of published studies have explored health care misinformation on social media, with four studies focusing explicitly on CAM misinformation. In regard to these four studies that specifically explored CAM misinformation on social media, one study looked into the use of social media in the promotion of alternative oncology and data about cancer [34]. The study found social media to be a useful channel for sharing patients' experiences with alternative oncology, but also an ideal environment for spreading false information. Moreover, one study identified Twitter users who were propagating information on CAM treatments claiming to treat or cure cancer and found that cancer treatment misinformation is frequently spread by actors other than patients [74]. Another study that evaluated how hypertension is portrayed on YouTube found that 33% of the videos were misleading and 70% of the misleading videos were about unproven alternative treatments [23]. A similar study that evaluated the reliability and quality of information in YouTube videos on traditional Chinese medicine and inflammatory arthritis found that almost half (46%) of included videos provided misleading information [75]. In regard to the studies that explored health misinformation on social media, one systematic review identified the main health misinformation topics and their prevalence across various social media platforms [76]. Health misinformation was the most prevalent on Twitter and YouTube and on issues related to vaccines and smoking products or drugs. Additionally, one pilot study tracked the sharing of posts containing health misinformation in the Polish language social media [77]. According to the study, roughly 40% of health information posts that were shared contained links that were identified as misinformation. Furthermore, in another study, researchers investigated the dissemination of gynecologic cancer-related misinformation on Weibo [78]. While the majority of gynecologic cancer-related tweets contained medically accurate information, almost 35% of them contained false or inaccurate information. Non-governmental organizations, and public health and government agencies have all been cited as critical in generating the fast response needed to communicate accurate information and rectify misinformation on social media [79–81].

#### Discussion of theme 3: Challenges with social media research in the context of CAM

A preliminary review of the literature found two studies that described the difficulties encountered while conducting health-related social media research. The first study looked at health-related misinformation on Twitter, specifically in relation to alternative medications that claim to treat or cure cancer, and found a multitude of challenges that limited the study's generalizability [74]. These challenges included the difficulty in detecting legitimate personal accounts (as opposed to bots or organizational accounts), accessibility issues such as a visual impairment or other constraints to engaging in social media, and the fact that some health conditions are associated with a social stigma, which may limit their discussion on social media. Another study that explored social media research in gastroenterology highlighted the challenges in reliability and ethical considerations [82]. Specifically, it discussed the excess amount of meaningless data such as data from bots and organizational accounts (e.g., pharmaceutical companies) which can compromise the reliability of results. Furthermore, the study discussed the ethical challenges of conducting social media research, particularly the threats to privacy and informed consent that can occur as data from subjects is often collected without the user's direct knowledge.

### Areas identified for further research

We have identified a few areas for future research based on our findings.

Currently, there exists more information on social media about the use of CAM, CAM products, and CAM adverse events than ever before, yet the quality of studies exploring social media research in the context of CAM is unknown [24, 83–86]. We hypothesize that this research gap can be explained based on a number of reasons, including a lack of academic research funding, the prioritization of conventional medicine research, as well as methodological and ethical obstacles which make it difficult to conduct high quality CAM research [87–90]. Specifically with regard to methodological obstacles, the physical nature of CAM therapies (e.g., massage therapy, acupuncture) makes it difficult for researchers to construct an acceptable placebo control [89]. With regard to the ethical challenges, informed consent is often difficult to obtain because in CAM-related research, patients are often predisposed to strongly prefer a CAM treatment over a control (placebo or non-CAM) treatment [89]. Patients, health care professionals, researchers, and policymakers all require reliable, credible, and up-to-date information as well as shared experiences and engagement with CAM [87–90]. This justifies a need for an updated review of social media research in the context of CAM along with a quality appraisal of relevant studies. In addition, while several published studies examined the efficacy of social media as a platform for delivering health care information, to our knowledge, there were none that measured the efficacy of social media as a platform for delivering CAM-related information. Thus, further research is needed to explore the efficacy of social media as a platform for delivering CAM-related information. Furthermore, in addition to future research continuing to examine social media platforms, patient-authored texts in online health forums and medical blogs could offer a valuable resource to further understand individuals’ attitudes and beliefs regarding CAM treatments [91, 92]. Additionally, if a critical appraisal tool is developed, a future direction could include critically appraising social media-related studies.

Moreover, research has shown that group polarization is prevalent on social media platforms involving controversial issues, which limits information dissemination among those with opposing views [93–97]. However, to our knowledge, it has not yet been explored as to whether this is also the case with CAM discussion on social media. If it is the case that the increasingly personalized algorithms on popular social media platforms expose individuals more often to posts that reinforce their beliefs and less often to posts containing novel information, it is possible that the confirmation bias is being magnified [98–101]. As an example, one study found that social media users who were exposed to health articles that conformed to their initial beliefs were more likely to share the article on social media [102]. Further research should explore the degree to which information is shared among dissimilar individuals on social media in the context of CAM [67, 94].

### Strengths and limitations

A main strength of the study includes the fact that multiple bibliographic databases were systematically searched, in addition to the grey literature. Further to this, the title and abstract screening, and data extraction were completed independently and in duplicate. Limitations of this study include the fact that only articles written in the English language were included, thus, important findings from non-English language articles may have been missed. Furthermore, while not a limitation in itself, many of the types of studies included in our review did not utilize commonly used research study designs for which a reporting guideline or validated quality appraisal tool was available (e.g., social media analyses), thus future work is warranted in this area. Additionally, CAM is an umbrella term that represents a very wide range of therapies that differ widely in nature. Thus, while our search strategy and the definition of CAM we used when determining article eligibility were comprehensive, certain types of CAM may have been missed. Similarly, many types of social media exist. Thus, while our search strategy likely captured the most prominent types, some forms of less well-known social media may have also been missed. We also acknowledge that because our searches were primarily restricted to biomedical and health databases, literature from the fields of marketing, strategic communication, anthropology, sociology, and medical humanities may not have been fully captured in this review.

## Conclusions

The present scoping review involved a systematic search of the literature to identify the quantity and type of studies investigating the ways in which social media is used in the context of CAM. From 29 eligible articles, we identified three major themes including: 1) social media is used to share user/practitioner beliefs, attitudes, and experiences about CAM, 2) social media acts as a vehicle for the spread of misinformation about CAM, and 3) there are unique challenges with conducting social media research in the context of CAM, specifically regarding collecting a representative sample of data. Additionally, we highlight that while a substantial number of articles are available to practitioners, patients, and researchers, the quality and update frequency for many of these articles vary widely, and until formally assessed, remain unknown. Furthermore, we identify that a need exists to conduct an updated and systematically searched review of CAM-related health care or research resources on social media.

## Data Availability

All relevant data are included in this manuscript.

## Abbreviations

CADTH: Canadian Agency for Drug and Technologies in Health
CAM: Complementary and alternative medicine
CBD: Cannabidiol
IBS: Irritable bowel syndrome
NCCIH: National Center for Complementary and Integrative Health
NICU: Neonatal intensive care unit
PTSD: Posttraumatic stress disorder
RCT: Randomized control trial
SMT: Spinal manipulation therapy
SSS: Severity scoring system
